# Biomolecular network-based synergistic drug combination discovery: a combination of paeoniflorin and liquiritin alleviates neuropathic pain by inhibiting neuroinflammation via suppressing the chemokine signaling pathway

**DOI:** 10.1038/s41392-020-0160-8

**Published:** 2020-05-22

**Authors:** Qiuyan Guo, Weijie Li, Chao Wang, Xia Mao, Xiaoyue Wang, Wenjia Chen, Haiyu Xu, Qian Wang, Yanqiong Zhang, Na Lin

**Affiliations:** 1 Institute of Chinese Materia Medica, China Academy of Chinese Medical Sciences, 100700 Beijing, China; 2 State Key Laboratory of Natural and Biomimetic Drugs, School of Pharmaceutical Sciences, Peking University, 100191 Beijing, China

**Keywords:** Target identification, Neuroimmunology

**Dear Editor**,

Neuropathic pain (NP) resulting from injuries or diseases affecting the somatosensory nervous system is highly prevalent in various pathological conditions.^1^ Since NP is debilitating and impacts health and the quality of life, there is an urgent need for effective non-addictive new therapies. Traditional Chinese medicine (TCM) has been increasingly used for its benefits in relieving pain in clinics. Wu-Tou Decoction (WTD), which was first described by the famous TCM classic “*Jin Gui Yao Lue”*, is one of the most effective TCM herbal prescriptions for pain management. A growing body of clinical evidence shows that WTD markedly alleviates different types of NP, such as trigeminal neuralgia, inflammatory pain, and cancer-induced pain, with a total effectiveness of ~80%.^2^ Our previous data demonstrated the analgesic effects of WTD via suppression of glial cell activation and neuroinflammation and further revealed that WTD attenuated NP partially by inhibiting spinal astrocytic IL-1R1/TRAF6/JNK signaling and regulating the glutamatergic system in CA3 in spinal nerve ligation (SNL)-induced NP in vivo.^3,4^ However, the bioactive compounds (BACs) of WTD that have an effect on NP and the underlying mechanisms remain unclear.

In this study, we identified a total of 77 chemical compounds in the aqueous extract of WTD by HPLC/ESI-LTQ-Qrbitrap-MS (Supplementary Table S1). Of these compounds, 43 were screened as candidate BACs with potential druggability by ADME evaluation in silico (Supplementary Table S2). To investigate the pharmacological effects of the candidate BACs of WTD, their putative targets were predicted. Interestingly, the candidate BACs in WTD shared 107 targets with 46 known FDA-approved analgesic agents (Supplementary Fig. S1a), implying their potential therapeutic efficiency for the treatment of NP. To illustrate the relationship of the putative targets of WTD with the known therapeutic targets of NP, the “NP-related genes-WTD putative targets” interaction network was constructed, and the 453 hubs, representing the most highly connected genes in the network, were screened. The hub subnetwork was further built, and the 130 major hubs were identified by ranking the hub importance according to the values of the network topological centralities (degree, betweenness, and closeness centralities). Functionally, the major hubs were found to play roles in various pathways related to neuroinflammation and neurotransmitter transportation (Fig. 1a and Supplementary Fig. S1b, c). Notably, six putative targets of WTD (CCL5, CCR5, GNAI1, SRC, PIK3CA, and AKT) are involved in the chemokine signaling pathway, which was the most significantly enriched pathway among the major hubs (Supplementary Fig. S1b). As illustrated in Fig. 1b and Supplementary Fig. S1d, spinal cord injuries may impair the binding of CCL5 with its receptor CCR5 to regulate GNAI1 and subsequently activate the phosphorylation of SRC and the downstream proteins PIK3CA and AKT, leading to neuroinflammation during NP development and progression. In this context, the CCL5-CCR5-GNAI1-SRC-PIK3CA-AKT axis was identified as a candidate target of WTD for the treatment of NP. To screen the major BACs that impact this axis, molecular docking was performed to simulate the binding ability and mode of WTD candidate BACs with the CCL5, CCR5, GNAI1, SRC, PIK3CA, and AKT proteins. According to the docking scores, both paeoniflorin (PAE) and liquiritin (LIQ) were demonstrated to be the BACs with the strongest binding to the candidate target proteins (Fig. 1c and Supplementary Fig. S2), which was further verified by surface plasmon resonance (SPR) assays (Fig. 1d and Supplementary Fig. S3a, b).

**Fig. 1:**
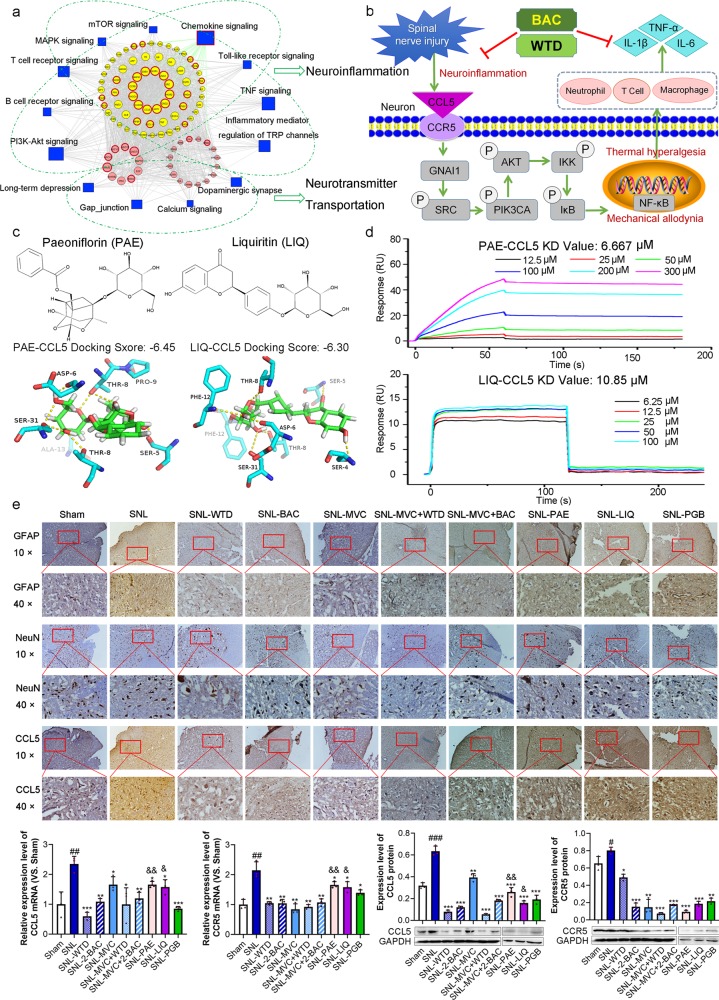
Biomolecular network-based synergistic drug combination discovery for pain control. **a** Interaction network of “NP-related genes-WTD candidate targets” illustrated according to the links between NP-related genes and WTD candidate targets. Rectangular nodes represent the NP-related pathways enriched with WTD candidate targets. Circular nodes represent the WTD candidate targets. Circular nodes with red highlights refer to the common targets of WTD and known FDA-approved analgesic agents. **b** Schematic diagram of the CCL5-CCR5-GNAI1-SRC-PIK3CA-AKT signaling axis with the candidate targets of WTD for the treatment of NP. **c** The chemical structures of PAE and LIQ and molecular docking simulations of their binding patterns with the corresponding target proteins with strong binding efficiency. **d** The binding affinity of PAE or LIQ with CCL5 was verified by an SPR assay. **e** The influence of the two-BAC combination on GFAP, NeuN, and CCL5 in the dorsal horn of L5 spinal cord tissues in SNL rats was examined by immunohistochemistry, and the regulatory effects of the two-BAC combination on the CCL5-CCR5-GNAI1-SRC-PIK3CA-AKT signaling axis at both the mRNA and protein levels was detected by qPCR and western blot analyses; the downstream neuroinflammation-related factors were detected by ELISA

The usage of paired or multiple drugs in combination to synergistically target multiple disease proteins or pathways has shown higher effectiveness compared to monotherapy. Hence, we investigated the pharmacokinetics and analgesic effects of the two-BAC combination of PAE and LIQ and compared them with those of WTD and PAE and LIQ alone. The results showed that the amount of time required to reach the maximum plasma concentration (T_max_) for both PAE and LIQ after the administration of the two-BAC combination was 5 min, which was less than that of WTD (120 min for PAE and 30 min for LIQ), suggesting the rapid absorption of the two-BAC combination (Supplementary Fig. S3c, d and Supplementary Table S3). The maximum plasma concentrations (C_max_) of PAE(668 ng/mL) and LIQ (17.1 ng/mL) in rats receiving the two-BAC combination were 4.64-fold and 1.29-fold higher, respectively, than those in rats receiving WTD (144 ng/mL for PAE and 13.3 ng/mL for LIQ). In addition, a SNL-induced NP rat model was established to evaluate the analgesic effects and to validate the pharmacological mechanisms of the two-BAC combination of PAE and LIQ. Compared with that of the sham group, SNL markedly reduced the 50% paw withdrawal threshold (PWT, *P* < 0.001), which was significantly increased by the administration of WTD and the two-BAC combination from 0.5 to 2.5 h and reached a peak at 1 h post drug administration (all *P* < 0.05, Supplementary Fig. S4a, b). Regarding cold hyperalgesia, SNL rats were more sensitive to cold stimuli than the rats in the sham group (*P* < 0.001, Supplementary Fig. S4a, b). The reaction times to cold stimuli of SNL rats were significantly reduced by treatment with WTD and the two-BAC combination (all *P* < 0.05, Supplementary Fig. S4a, b). Moreover, morphological characterization of immunohistochemically identified astrocytes and neurons in spinal cord tissues according to the specific markers GFAP and NeuN demonstrated that the administration of both WTD and the two-BAC combination effectively inhibited the activation of astrocytes in SNL rats (all *P* < 0.05, Fig. 1e and Supplementary Fig. S5), implying that the drug treatments might inhibit neuroinflammation during NP progression. Importantly, the intervention capability of the two-BAC combination for mechanical allodynia, cold hyperalgesia and neuroinflammation showed no significant difference compared to those of WTD, and the administration of PAE and LIQ alone resulted in less prominent therapeutic effects than that of the two-BAC combination (all *P* < 0.05, Supplementary Fig. S4a, b). Furthermore, we evaluated the indexes of the heart, liver, kidney, lung, and spleen of the rats in the different groups. As shown in Supplementary Fig. S6, SNL decreased the spleen index, which could be reversed by treatment with WTD, the two-BAC combination, MVC, a combination of WTD and MVC, and a combination of the two BACs and MVC. There were no significant differences in the indexes of the heart, liver, kidney, and lung among the different groups. The above data suggest that treatment with WTD and the two-BAC combination have no apparent side effects in rats.

Growing evidence shows that targeting the chemokine signaling pathway may be crucial in neuropathy control.^5^ After determining the analgesic effects of the two-BAC combination of PAE and LIQ, a series of molecular biological experiments verified that it significantly suppressed CCL5, CCR5, GNAI1, SRC, PIK3CA, and AKT expression at both the mRNA and protein levels in spinal cord tissues and subsequently reduced the serum levels of the neuroinflammatory factors TNF-α, IL-1β, and IL-6 (Fig. 1e and Supplementary Figs. S7 and 8). Furthermore, we also compared the analgesic effects and regulatory mechanisms of WTD and the two-BAC combination with those of MVC (maraviroc, an antagonist of CCR5), the MVC-two-BAC combination and the MVC-WTD combination. The data shown in Fig. 1e and Supplementary Figs. S4–8 indicated that both WTD and the two-BAC combination exerted effects similar to those of MVC. In contrast, single intrathecal injection of a ligand of CCR5 (macrophage inflammatory protein-1α, MIP) in naïve rats evoked hypersensitivity to mechanical and cold stimuli, which were significantly attenuated by the administration of WTD and the two-BAC combination (Fig. 1e and Supplementary Figs. S4–8), implying that both WTD and the two-BAC combination might function as antagonists of CCR5.

In summary, PAE and LIQ may be the main BACs in WTD that alleviate NP by inhibiting neuroinflammation through reducing the expression and activity of the CCL5-CCR5-GNAI1-SRC-PIK3CA-AKT signal axis, which is the major component of the chemokine signaling pathway. The combination of PAE and LIQ may be a promising therapeutic agent for pain control.

## Supplementary information

Supplementary files

Fig.S1

Fig.S2

Fig.S3

Fig.S4

Fig.S5

Fig.S6

Fig.S7

Fig.S8

raw data of Fig.S3c and Fig.S3d

raw data of Fig.S4a and Fig.S4c
