# Supplementary material for: Biomolecular network-based synergistic drug combination discovery: a combination of paeoniflorin and liquiritin alleviates neuropathic pain by inhibiting neuroinflammation via suppressing the chemokine signaling pathway
Source: Signal Transduct Target Ther. 2020 May 22;5:73. doi: 10.1038/s41392-020-0160-8 (PMC7242454; doi:10.1038/s41392-020-0160-8)
Supplement: Supplementary file 1 — Supplementary files [file 41392_2020_160_MOESM1_ESM.docx]

**Supplementary Files for**

**A Biomolecular Network-based Synergistic Drug Combination Discovery: Paeoniflorin and Liquiritin Combination Alleviates Neuropathic Pain by Inhibiting Neuroinflammation via Suppressing Chemokine Signaling Pathway**

Qiuyan Guo^1^, Weijie Li^1^, Chao Wang^1^, Xia Mao^1^, Xiaoyue Wang^1^, Wenjia Chen^1^, Haiyu Xu^1^, Qian Wang^2^, Yanqiong Zhang^1,^*, Na Lin^1,^*

**Correspondence to:** yqzhang@icmm.ac.cn & nlin@icmm.ac.cn

**Materials and methods**

**Supplementary Tables**

**Supplementary Figure legends**

**Materials and methods**

*Chemical profiling of WTD and ADME evaluation in silico*

The chemical compounds of the five herbs contained in the water extract of WTD were detected by HPLC/ESI-LTQ-Orbitrap-MS. The water extract of WTD was made according to the quality control standards of the China Pharmacopoeia, centrifuged at 4000 rpm for 5min, then the supernatant was filtered through a 0.22μm filter.After that, the filtrate was injected into the HPLC-MS system. The analysis of the chemical compounds of WTD was performed with a Thermo scientific HPLC system coupled with a LTQ Orbitrap XL instrument (Thermo scientific, U.S.A) and the separation was conducted on a Kromasil chromatographic column (250 mm×4.6 mm, 5 μm, Amsterdam, Netherlands) and a precolumn Sweden AKZO-NOBEL (Amsterdam, Netherlands). Formic acid aqueous solution (0.01% v/v, solvent A) and methanol (solvent B) were used as mobile phases. Gradient elution was performed as follows: 0-40 min, 95-55% (A); 40-60 min, 55-0%(A) at a flow-rate of 1.0 mL/min and injection volume was 10 μL. The MS analysis was accomplished using the ESI sources in both negative and positive ion mode. For positive ion mode, the parameters were set as follows, the spray and capillary voltage was 4 KV and 25 V respectively, the vaporizer and capillary temperature was 300℃ and 350℃ respectively, the flow rates of sheath gas (high purity nitrogen) and aux gas (high purity helium) was 30 L/min and 5 L/min respectively, the tube lens voltage was 110 V. For negative ion mode, the parameters were: the spray and capillary voltage was −3 KV and −35V respectively, the vaporizer and capillary temperature was 300℃ and 350℃ respectively, the flow rates of sheath gas and aux gas was 30 L/min and 5 L/min respectively, the tube lens voltage was -110 V, and full-mass range was 100-1000 Da.

The structural information (*.mol or *.sdf files) of chemical compounds were downloaded from ChemSpider (http://www.chemspider.com/) and the ADME evaluation of the above compounds carried out using ACD/Percepta software 5.07 (ACD/Labs, Toronto, Canada), including the passive intestinal permeability of Caco-2 module and the PK explorer module to predict their oral bioavailability.

*Collection of known NP-related genes*

Known NP-related genes were collected from DrugBank database (http://www.drugbank.ca/, version: 5.0), Human Phenotype Ontology(https://hpo.jax.org/, June 2018 release), Therapeutic Target Database (TTD, http://bidd.nus.edu.sg/group/cjttd/, Sep 15th, 2017) and DisGeNET-a database of gene-disease associations(http://www.disgenet.org/, v6.0). Collectively, a total of 426 known NP-related genes were used in the following sections (**Supplementary Table S2**).

*Construction of the "NP-related gene--WTD-putative target" interaction network*

The "NP-related gene--WTD-putative target" interaction network was constructed using the links between the known NP-related genes and the putative targets of the chemical compounds identified in the water extract of WTD, which were obtained from the public database STRING(Search Tool for Known and Predicted Protein-Protein Interactions, version 10.0, http://string-db.org/). Navigator software (Version 2.2.1) was used for the network visualization. The values of three topological features, including node's degree, betweenness and closeness, were calculated to evaluate the topological importance of the nodes in the network and chose the major network targets.For each node *i* in interaction network, we defined three measures for assessing its topological property: (1) 'Degree' is defined as the number of links to node *i*; (2) 'Node betweenness' is defined as the number of shortest paths between pairs of nodes that run through node *i*. (3)‘Closeness’is defined as the inverse of the farness which is the sum of node *i* distances to all other nodes. The Closeness centrality can be regarded as a measure of how long it will take to spread information from node *i* to all other nodes sequentially. Degree,node betweenness and closeness centralities can measure a node’s topological importance in the network. The larger a node’s degree/ node betweenness /closeness centrality is, the more important the node is in the interaction network.

*Pathway enrichment analysis*

Kyoto Encyclopedia of Genes and Genomes (KEGG) pathway enrichment analysis was operated based on the Database for Annotation, Visualization and Integrated Discovery (DAVID, http://david.abcc.ncifcrf.gov/home.jsp, version 6.7) and pathway data which were collected from the FTP service of KEGG (http://www.genome.jp/kegg/, last updated: Oct 26, 2018). In this study, we selected KEGG pathways with P-values <0.05 (corrected using the Bonferroni method).

*Molecular Docking*

Molecular docking simulation was performed to calculate the binding efficiency of BAC and the corresponding target proteins by Ledock (http://www.lephar.com/, version 1.0) and Pymol (https://pymol.org/2/, version 1.7.2.1). The protein structure information of the candidate targets of WTD was collected from RCSB protein data bank (www.pdb.org, Updated in 2016-5) and their resolutions have been carefully checked. The 3D structures (*.mol files) of PAE and LIQ were prepared by ChemDraw. The docking score (kcal/mol) indicates the binding energy between the ligand and the receptor. The higher absolute value of docking score represents the stronger binding efficiency of BAC and the corresponding target proteins.

*Surface Plasmon Resonance (SPR) experiments*

Biacore T200 was used to measure the direct binding affinities between compounds and CCL5 (GE healthcare). CCL5 was immobilized on a CM5 sensor chip by using standard amine-coupling at 25℃ with running buffer HBS-P (20 mM HEPES buffer, 2.7 mM NaCl, 137 mM KCl, 0.05% surfactant P-20, pH 7.4). CCL5 concentration was fixed at 30 ng/µL and the immobilization level of GLU was about 8000 RU. Different concentrations of compounds containing 5% DMSO were serially injected into the channel to evaluate the binding affinity. A reference channel was only activated and blocked to eliminate compound unspecific binding to the surface of the chip. Regeneration buffer of 10 mM NaOH was added after each cycle of PAE. Extra wash with 30% DMSO was also added to remove the last remaining sample in the pipeline. The association constants (ka), the dissociation constants (kd) and the equilibrium dissociation constants (KD) of the compounds were obtained by fitting the data sets to 1:1 Langmuir binding model using Biacore T200 Evaluation Software.

*Experimental validations*

Animals, drugs and experiment design

Male Sprague Dawley rats (n=50, 180-200 g) were purchased from Beijing Vital River Laboratory Animal Technology Ltd with the production license No of SYXK 2015-0041. All animals were maintained in a room at a constant temperature of 24 ± 1°C with a 12-hour light/dark cycle, in addition, they were allowed free access to food and water.

Drugs used in this study were as follows: (1) The water extract of WTD was prepared according to the quality control standards of the China Pharmacopoeia, and then made into lyophilized powder (1g WTD lyophilized powderwas equal to 3.71g raw medicinal herbs of WTD). The dosage of WTD was 15 g/(kg·day) at the concentration of 0.75 g/mL, which was equivalent to the four folds of patient's daily dosage in clinics. (2) BAC was prepared according to their contents in the freeze-dried powder of WTD (PAE: 9.225 µg/mg;LIQ: 3.740 μg/mg) detected by the LC-MS analysis. As the dosage administration of WTD was 0.75 g/mL, a two-BAC-combination consisting of 1865.25 μg/mL PAE and 756 μg/mL LIQ were formed. PAE (CAS: 23180-57-6) and LIQ CAS: 551-15-5) were purchased from Shanghai Standard Technology Co., Ltd. with the purity of HPLC/ESI-LTQ-Orbitrap-MS≥ 98%. (3) MVC (Selleckchem, Houston, TX, USA) was dissolved in 12% DMSO to the concentration of 4 μg/μL and was administrated to every rat 5 μL by intrathecal injection as described in the previous study.(4) MIP-1α (Rat Recombinant CCL3 Protein, Sino Biological) was used at the concentration of 10 pM by intrathecal injection as described in the previous study.Animals in the Sham and SNL groups were administered an equal volume of distilled water.

To validate the analgesic effects of the PAE andLIQ combination and its regulatory effects on the candidate targets, we performed two parts of*in vivo*experiments. In the first part, a total of 70 rats were randomly divided into seven groups (n=10, per group) includingthe Sham, SNL, WTD-treatment, the two-BAC combination-treatment, MVC-treatment, MVC+WTD-treatment and MVC+two-BAC-treatment groups.The drug treatment was performed for three days via oral administration starting on the fourth day after SNL or Sham operation. In the second part, a total of 40 rats were randomly divided into four groups (n=10, per group) including the Sham, MIP-treatment, MIP+WTD-treatment and MIP+two-BAC-treatment groups.The drug treatment was performed for three days after the implantation of the intrathecal catheter.

Inductionof SNL model

SNL rat model was established by the modified method as described in the previous study.In brief, rats were anesthetized via isoflurane inhalation anesthesia.Then, the transverse process of the sixth left lumbar was removed to expose the L5 spine nerve.After that, the L5 nerve was carefully separated and tightly ligated with a surgical suture, followed by washing the skin with normal saline and sutured. The L5 spine nerve of the Sham rats were kept in-ligatured.

Measurement of mechanical hypersensitivity

Rats were acclimatized in individual clear boxes on the wire-mesh platform, to allow access to the ventral surface of the hind paws.The mechanical hypersensitivity was assessed by the sensitivity to the application of von Frey hairs (Stoelting Co., Chicago, USA). The von Frey filaments (1.0–100.0 g) were presented perpendicularly to the plantar surface of the injected paw and held in this position for 5–8 s with enough force to cause a slight bend in the filament. Positive responses included an abrupt withdrawal of the hind paw or flinching behavior immediately following removal of the stimulus. A median paw withdrawal threshold was determined using an adaptation of Dixon's up-down method. The 50% paw withdrawal threshold was analyzed at 0, 0.5, 1, 1.5, 2, 3h and 2, 3 days after drugs administration, respectively.

Measurement of cold hypersensitivity

Briefly, 20 μL acetone was sprayed gentlyto the middle of the plantar surface of the hind paw by a 1 mL syringefrom a short distance. The amount of times that rats were licking and/or shaking the hind paw during 5 min after acetone applicationwas recordedand used as an index of responsiveness to cold hypersensitivity. The licking and/or shaking times was measured at 0, 0.5, 1, 1.5, 2, 3 h and 2, 3 days after drugs administration, respectively.

The implantation of the intrathecal catheter

Firstly, the rat was deeply anesthetized and then accepted the laminectomy to removethe thoracic spine. Then, the polyethylene drain was inserted into the spinal cord and located at the L4 to L5 level. The next, to judge whether the location of the PE drain was appropriate, 5 µL lidocaine (200 mg in 10 mL) was injected into the intrathecal catheterfollowed by saline, and the hind legs of rats were completely paralyzed post the administration of lidocaine was regarded as the positive signs. Rats without evidence of neurologic deficit or paralysis after the surgery and lidocaine injection were selected for further experiments in this study.

Immunohistochemistry

Immunohistochemistry was performed to detect the expression patterns and cellular localization of GFAP, NeuN and CCL5 proteins in the dorsal horn of theL5 region of the rat spinal cordaccording to the previous descriptions. In this study, anti-GFAP antibody (1:100, abcam, ab10062), anti-NeuN antibody (1:100, abcam, ab104224) and CCL5 polyclonal antibody (1:100, Elabscience, E-AB-17864) were used as the primary antibodies, a horseradish peroxidase (HRP) conjugated secondary antibody were used for half an hour at room temperature and diamino benzidine (DAB) was used as a substrate.

qRT-PCR analysis

qRT-PCR analysis was performed to detect the gene expression levels of CCL5, CCR5, GNAI1, SRC, PIK3CA and AKT. GAPDH was used as the internal control for normalization and quantification of CCL5, CCR5, GNAI1, SRC, PIK3CA and AKT expression. The primer sequences were listed as following table.

| CCL5-Forward | TGCCCACGTCAAGGAGTATTT |
| --- | --- |
| CCL5-Reverse | TCTCTGGGTTGGCACACACTT |
| CCR5-Forward | TCATAAAACCAGTGTGAAACAAATTG |
| CCR5-Reverse | TCATGTTACCCACAAAACCAAAGA |
| GNAI1-Forward | TGCAAGCCTGCTTCAACAGA |
| GNAI1-Reverse | AAGTCATTCAGGTAGTACGCCG |
| SRC-Forward | GGACAGTGGCGGATTCTACATC |
| SRC-Reverse | AGCTGCTGCAGGCTGTTGA |
| PIK3CA-Forward | AGATGTGGACACCCAAGCTG |
| PIK3CA-Reverse | GCAGGGAGTCAAAATACCCACT |
| AKT2-Forward | AGAGAGCCGAGTCCTACAGAATA |
| AKT2-Reverse | CCGAGAGAGGTGGAAAAACA |
| GAPDH-Forward | GGCACAGTCAAGGCTGAGAATG |
| GAPDH-Reverse | ATGGTGGTGAAGACGCCAGTA |

Relative quantification of mRNA expression was evaluated by the comparative cycle threshold (CT) method, all experiments were performed in triplicate.

Western blot analysis

Western blot analysis was performed to detect the protein expression levels of CCL5, CCR5, GNAI1, p-SRC, p-PIK3CA and p-AKT in the dorsal horn of L5 spinal cord tissue. The detailed information of the antibodies used in this study was provided as following table.

| **Name** | **Concentration** | **Company and product NO.** |
| --- | --- | --- |
| anti-CCL5 | 1:500 | ab9783 (Abcam) |
| anti-CCR5 | 1:500 | YT6108 (Immunoway) |
| anti-GNAI1 | 1:1000 | 12617-1-AP (Proteintech Group) |
| anti-p-SRC | 1:1000 | 12432s (CST) |
| anti-p-PIK3CA | 1:250 | orb6718 (biorbyt) |
| anti-p-AKT | 1:1000 | 4060s (CST) |
| anti-GAPDH | 1:10000 | abcam8245(Abcam) |

All experiments were performed in triplicate.

ELISA

ELISA was performed to measure the serum levels of tumor necrosis factor-alpha (TNF-α), interleukin (IL)-1β, IL-6 in different groups. ELISA kits were purchased from Elabscience Biotechnology Co, Ltd (Wuhan, China) and all experiments were carried out according to the manufacturer's protocols and the results were measured at 450 nm.

*Statistical analysis*

Statistical analyses were performed using SPSS statistical software (Version 19.0). For all quantification analyses, one-way ANOVA analyses were used. *P*-values less than 0.05 were considered significant. Data were represented as the mean ± s.

**Supplementary Tables**

**Table S1. Characterization of 77 chemical constituents of WTD and five single herb by HPLC-ESI-LTQ-MS**

| NO | Rt | Identification | Negative (m/z） | | Positive (m/z) | | Element  composition | Mv  (Da) | MS/MS (m/z) | Papp  (10^-6^ cm/s) | OB  (%F) | source |
| --- | --- | --- | --- | --- | --- | --- | --- | --- | --- | --- | --- | --- |
|  |  |  | Indicated | ppm | Indicated | ppm |  |  |  |  |  |  |
| 1 | 5.68 | Paeonilactone B | 195.07 | 4.12 | 197.08 | 2.31 | C_10_H_12_O_4_ | 196 | 151.02[M-H-CO_2_]^-^;  150.91[M+H-CO_2_H_2_]^+^ | 53.0 | 99.8 | Ⅱ |
| 6 | 36.88 | Galloylpaeoniflorin | 631.17 | 0.06 | 655.16 | 2.71 | C_30_H_32_O_15_ | 632 | 613.33[M-H-H_2_O]^-^ ;  341.16[M+Na-C_12_H_7_O_8_]^+^ | 9.0 | 96.4 | Ⅱ |
| 8 | 46.45 | Benzoylpaeoniflorin  Sulfonate | 647.14 | 0.59 | — | — | C_30_H_32_O_14_S | 648 | — | 40.0 | 99.7 | Ⅱ |
| 9 | 50.13 | Benzoylpaeoniflorin | 629.19 | 0.27 | 585.19 | 3.44 | C_30_H_32_O_12_ | 584 | 583.20[M+CHO_2_-H]^-^ ;  319.14[M+H-C_16_H_11_O_3_]^+^ | 40.0 | 99.7 | Ⅱ |
| 10 | 4.33 | Gancaonin D | 383.12 | 15.60 | — | — | C_21_H_20_O_7_ | 384 | 341.17[M-H-C_2_H_2_O]^-^;  323.16[M-H-C_2_H_4_O_2_]^-^； | 22.0 | 91.9 | Ⅲ |
| 13 | 18.19 | Glycyrrhisoflavone/ /Licoisofiavanone/ Licoisofiavone | 353.10 | 7.62 | — | — | C_20_H_18_O_6_ | 354 | 186.96[M-H-C_7_H_5_O]^-^;  164.95[M-H-C_13_H_16_O]^-^； | 23.0/146.0/20.0 | 85.3/81.4/90.2 | Ⅲ |
| 14 | 20.93 | Licoflavone/Inflacoumarin A /Glabrene | 321.11 | 11.9 | — | — | C_20_H_18_O_4_ | 322 | — | 197.0/145.0/136.0 | 64.7/65.2/65.2 | Ⅲ |
| 20 | 36.26 | Liquirtigenin | 255.07 | 0.14 | 257.08 | 3.4 | C_15_H_12_O_4_ | 256 | 134.80[M-H-C_4_H_8_O_4_]^-^ | 174.0 | 99.2 | Ⅲ |
| 21 | 36.81 | Pinocembrin | — | — | 257.08 | 3.17 | C_15_H_12_O_4_ | 256 | — | 195.0 | 98.2 | Ⅲ |
| 29 | 4.67 | Isomucronulatol | — | — | 303.13 | 1.5 | C_10_H_22_O_10_  _C17H18O5_ | 302 | — | 188.0 | 98.6 | Ⅳ |
| 30 | 20.49 | Rhamnocitin/ isomeride | 299.06 | 1.57 | — | — | C_16_H_12_O_6_ | 300 | — | 18.0/18.0 | 99.2/99.2 | Ⅳ |
| 31 | 35.71 | Prunetin/ isomeride | 283.06 | 1.59 | 285.07 | 3.54 | C_16_H_12_O_5_ | 284 | 267.98[M-H-CH_3_]^-^ ;270.05[M+H-CH_3_]^+^;253.08[M+H-CH_4_O]^+^; 225.03[M+H-C_2_H_4_O_2_]^+^ | 94.0/94.0 | 97.5/97.5 | Ⅳ |
| 32 | 37.94 | 2-Hydroxy-3-methoxystrychnine | 379.17 | 0.11 | — | — | C_22_H_24_N_2_O_4_ | 380 | 297.16[M-H-C_5_H_8_N]^-^ | 13.0 | 78.0 | Ⅳ |
| 33 | 38.43 | Rhamnocitin/ isomeride | 299.06 | 2.17 | — | — | C_16_H_12_O_6_ | 300 | 283.94[M-H-CH_3_]^-^ | 18.0/18.0 | 99.2/99.2 | Ⅳ |
| 34 | 43.57 | Prunetin/ isomeride | 283.06 | 2.76 | 285.07 | 2.81 | C_16_H_12_O_5_ | 284 | 267.93[M-H-CH_3_]^-^ ;  270.13[M+H-CH_3_]^+^;  253.03[M+H-CH_4_O]^+^ | 94.0/94.0 | 97.5/97.5 | Ⅳ |
| 35 | 45.81 | Methylnissolin | 299.09 | 1.44 | 301.11 | 3.22 | C_17_H_16_O_5_ | 300 | 284.04[M-H-CH_3_]^-^ ;  269.05[M+H-CH_4_O]^+^ | 224.0 | 69.4 | Ⅳ |
| 37 | 49.48 | Calycosin* | 283.06 | -0.35 | 285.08 | 2.28 | C_16_H_12_O_5_ | 284 | 268.05[M-H-CH_3_]^-^ ;  270.02[M+H-CH_3_]^+^;  253.06[M+H+CH_4_O]^+^ | 106.0 | 96.5 | Ⅳ |
| 38 | 55.42 | Formononetin* | 267.07 | 1.70 | — | — | C_16_H_12_O_4_ | 268 | 251.95[M-H-CH_3_]^-^ | 191.0 | 87.2 | Ⅳ |
| 39 | 9.11 | 1,2-benzenedicarboxylic acid, bis(2-methyl- propyl) ester | 277.09 | 2.00 | — | — | C_11_H_18_O_8_ | 278 | 114.90[M-H-C_6_H_10_O_5_]^-^ | 244.0 | 97.2 | Ⅴ |
| 44 | 17.47 | 1, 2- benzenedicarboxylic acid,bis(2-methylpropyl) ester | 277.13 | 2.96 | 279.14 | 7.96 | C_15_H_20_O_4_N | 278 | 235.01[M-H-C_2_H_2_O]^-^ ;  219.08[M+H-C_2_H_4_O_2_]^+^;  262.13[M+H-OH]^+^;  200.97[M+H-C_6_H_7_]^+^; | 224.0 | 99.8 | Ⅴ |
| 45 | 21.01 | Leucopelargonidin | 289.07 | 2.30 | — | — | C15H14O6 | 290 | 245.00[M-H-CO2]-； | 2.0 | 64.6 | Ⅴ |
| 46 | 21.75 | Dimethyl phthalate | 193.05 | 4.38 | — | — | C_10_H_10_O_4_ | 194 | 148.97[M-H-C_2_H_5_O]^-^; | 220.0 | 99.9 | Ⅴ |
| 48 | 28.09 | Cinnamic acid isomeride | 147.05 | 7.07 | — | — | C_9_H_8_O_2_ | 148 | 102.92[M-H-CO_2_]^-^ | 27.0 | 99.1 | Ⅴ |
| 49 | 28.86 | 2,3,5- trimethoxytoluene | 181.09 | 5.47 | — | — | C_10_H_14_O_3_ | 182 | 121.93[M-H-C_3_H_7_O]^-^  136.90[M-H-CO_2_]^-^ | 220.0 | 99.6 | Ⅴ |
| 50 | 29.5 | Isobutyl benzoate | 223.10 | 2.98 | — | — | C_11_H_14_O_2_ | 224 | 208.04[M+CHO_2_-CH_3_]^-^;  178.97[M+CHO_2_-CO_2_]^-^ | 245.0 | 99.8 | Ⅴ |
| 52 | 33.73 | (+)-syringaresinol | 417.16 | 1.45 | — | — | C_22_H_26_O_8_ | 418 | 180.92[M-H-C_12_H_12_O_5_]^-^；  402.20[M+H-CH_3_]^-^ | 77.0 | 99.2 | Ⅴ |
| 53 | 36.75 | Dimethyl phthalate | 193.05 | 5.36 | — | — | C_10_H_10_O_4_ | 194 | 148.94[M-H-CO_2_]^-^;  177.99[M-H-CH_3_]^-^;  133.98[M-H-C_3_H_7_O]^-^ | 220.0 | 99.9 | Ⅴ |
| 55 | 39.24 | (+)-syringaresinol | 417.16 | 1.45 | — | — | C_22_H_26_O_8_ | 418 | 181.01[M-H-C_14_H_20_O_3_]^-^;  402.17[M-H-CH_3_]^-^ | 77.0 | 99.2 | Ⅴ |
| 57 | 42.31 | Llicoflavonol /Isolicoflavonol | — | — | 355.17 | 2.38 | C_16_H_28_O_7_ | 354 | 266.98[M+Na-C_4_H_24_O]^+^;  285.01[M+Na-C_3_H_18_O]^+^ | 21.0/21.0 | 85.6/85.6 | Ⅴ |
| 58 | 49.47 | Cinnamic acid * | 147.05 | 7.44 | — | — | C_9_H_8_O_2_ | 148 | 102.92[M-H-CO_2_]^-^ | 27.0 | 99.1 | Ⅴ |
| 59 | 57.61 | Trans-2-Nonenal | 139.11 | 7.90 | — | — | C_9_H_16_O | 140 | 70.72[M+H-C_5_H_8_]^-^;  110.93[M+H-CO]^-^ | 243.0 | 99.7 | Ⅴ |
| 63 | 16.75 | Talatizidine | — | — | 408.27 | 3.79 | C_23_H_37_O_5_N | 407 | — | 20.0 | 87.7 | Ⅵ |
| 67 | 22.7 | Talalisamine | — | — | 422.29 | 3.63 | C_24_H_39_NO_5_ | 421 | — | 20.0 | 87.7 | Ⅵ |
| 69 | 33.11 | 14-benzoyl-10-OH-mesaconine | 650.28 | 1.95 | 606.29 | 2.62 | C_31_H_43_O_11_N | 605 | 633.25[M+CHO_2_-OH]^-^ ;  556.39[M+H-C_2_H_2_N];  524.41[M+H-C_5_H_8_N];  588.48[M+H-H_2_O]^+^ | 8.0 | 96.4 | Ⅵ |
| 70 | 36.04 | 14-benzoyl-10-OH-aconine | 664.30 | 2.83 | 620.30 | 6.05 | C_32_H_45_O_11_N | 619 | 633.11[M+CO_2_H-CH_3_O]^-^  570.43[M+H-C_4_H_2_]^+^ | 7.0 | 95.5 | Ⅵ |
| 71 | 38.89 | Benzoylmesaconine* | 634.29 | 0.82 | 590.29 | 5.39 | C_31_H_43_NO_10_ | 589 | 616.38[M+CHO_2_-H_2_O]^-^;  588.45[M+CHO_2_-CH_2_O_2_]^-^  540.43[M+H-C_4_H_2_];  558.43[M+H-CH_4_O]^+^ | 13.0 | 98.3 | Ⅵ |
| 72 | 40.32 | Benzoylaconitine* | — | — | 604.31 | 4.11 | C_32_H_45_NO_10_ | 603 | 554.27[M+H-C_4_H_2_]^+^;  572.45[M+H-CH_4_O]^+^ | 22.0 | 99.3 | Ⅵ |
| 73 | 41.92 | Benzoylhypacoitine* | 618.29 | 0.004 | 574.30 | 4.53 | C_31_H_43_NO_9_ | 573 | 586.17[M+CHO_2_-CH_4_O]^-^ ;  524.41[M+H-C_4_H_2_]^+^;  542.42[M+H-CH_4_O]^+^ | 78.0 | 99.7 | Ⅵ |
| 74 | 42.76 | Benzoyldeoxyaconine | 632.31 | 4.11 | 588.31 | 3.52 | C_32_H_45_O_9_N | 587 | 614.32[M+CHO_2_-H_2_O]^-^ ;  556.44[M+H-CH_4_O]^+^ | 19.0 | 98.6 | Ⅵ |
| 75 | 43.52 | Beiwutine/10-OH-mesaconitine | — | — | 648.30 | 2.44 | C_33_H_45_NO_12_ | 647 | 588.47[M+H-C_2_H_4_O_2_]^+^ | 9.0/14.0 | 96.4/98.5 | Ⅵ |
| 76 | 46.28 | Hypaconitine* | — | — | 616.31 | 4.62 | C_33_H_45_NO_10_ | 615 | 556.40[M+H-C_2_H_4_O_2_]+;  524.46[M+H-C_7_H_8_]^+^ | 1250 | 99.7 | Ⅵ |
| 77 | 46.28 | Aconitine* | — | — | 646.32 | 2.91 | C_34_H_47_NO_11_ | 645 | 566.43896[M+H-C_5_H_5_O]^+^ | 45.0 | 99.6 | Ⅵ |

Ⅱ: Raidix Paeoniae Alba (Bai Shao), Ⅲ: Radix Glycytthizae (Gan Cao),Ⅳ:Radix Astragali (Huang Qi), Ⅴ: Herba Ephedrae (Ma Huang), Ⅵ: Radix Aconiti (Wu Tou), *: Being identified with Standard substance

# Table S2. Characterization of 43 candidate bioactive compounds of WTD

| Negative(m/z） | | | | | Positive(m/z) | | Element composition |  |  | Papp (10^-6^  cm/s) |  |  |
| --- | --- | --- | --- | --- | --- | --- | --- | --- | --- | --- | --- | --- |
| NO | Rt | Identification | Indicated | ppm | Indicated | ppm |  | Mv  (Da) | MS/MS (m/z) |  | OB  (%F) | sourc  e |
| 1 | 5.68 | Paeonilactone B | 195.07 | 4.12 | 197.08 | 2.31 | C10H12O4 | 196 | 151.02[M-H-CO_2_]^-^;  150.91[M+H-CO_2_H_2_]^+^ | 53.0 | 99.8 | Ⅱ |
| 2 | 30.71 | Paeoniflorin* | 525.16 | 1.36 | 503.15 | 5.85 | C23H28O11 | 480 | 449.18[M-H-CH2O]^-^；  479.13[M-H]^-^ ;  341.14[M+Na-C_7_H_7_O_3_]^+^;  381.19[M+Na-C_7_H_15_]^+^ | 1.0 | 44.7 | Ⅱ |
| 3 | 36.88 | Galloylpaeoniflorin | 631.17 | 0.06 | 655.16 | 2.71 | C30H32O15 | 632 | 613.33[M-H-H_2_O]^-^ ;  341.16[M+Na-C_12_H_7_O_8_]^+^ | 9.0 | 96.4 | Ⅱ |
| 4 | 46.45 | Benzoylpaeoniflorin  Sulfonate | 647.14 | 0.59 | — | — | C30H32O14S | 648 | — | 40.0 | 99.7 | Ⅱ |
| 5 | 50.13 | Benzoylpaeoniflorin | 629.19 | 0.27 | 585.19 | 3.44 | C30H32O12 | 584 | 583.20[M+CHO_2_-H]^-^ ;  319.14[M+H-C_16_H_11_O_3_]^+^ | 40.0 | 99.7 | Ⅱ |
| 6 | 4.33 | Gancaonin D | 383.12 | 15.60 | — | — | C21H20O7 | 384 | 341.17[M-H-C_2_H_2_O]^-^;  323.16[M-H-C_2_H_4_O_2_]^-^； | 22.0 | 91.9 | Ⅲ |
| 7 | 18.19 | Glycyrrhisoflavone/  /Licoisofiavanone/ Licoisofiavone | 353.10 | 7.62 | — | — | C20H18O6 | 354 | 186.96[M-H-C_7_H_5_O]^-^;  164.95[M-H-C_13_H_16_O]^-^； | 23.0/146.  0/20.0 | 85.3/81.4  /90.2 | Ⅲ |
| 8 | 20.93 | Licoflavone/Inflacouma rin A /Glabrene | 321.11 | 11.9 | — | — | C20H18O4 | 322 | — | 197.0/14  5.0/136.0 | 64.7/65.2  /65.2 | Ⅲ |
| 9 | 36.26 | Liquiritin* | 417.12 | 1.46 | 441.11 | 4.29 | C21H22O9 | 418 | 255.03[M-H-C_7_H_14_O_4_]^-^;  321.18[M+Na-C_4_H_8_O_4_]^+^;  423.27[M+Na-H2O]^+^ | 1.0 | 62.5 | Ⅲ |
| 10 | 36.26 | Liquirtigenin | 255.07 | 0.14 | 257.08 | 3.4 | C15H12O4 | 256 | 134.80[M-H-C_4_H_8_O_4_]^-^ | 174.0 | 99.2 | Ⅲ |
| 11 | 36.81 | Pinocembrin | — | — | 257.08 | 3.17 | C15H12O4 | 256 | — | 195.0 | 98.2 | Ⅲ |
| 12 | 4.67 | Isomucronulatol | — | — | 303.13 | 1.5 | C10H22O10 C17H18O5 | 302 | — | 188.0 | 98.6 | Ⅳ |
| 13 | 20.49 | Rhamnocitin/ isomeride | 299.06 | 1.57 | — | — | C16H12O6 | 300 | — | 18.0/18.0 | 99.2/99.2 | Ⅳ |
| 14 | 35.71 | Prunetin/ isomeride | 283.06 | 1.59 | 285.07 | 3.54 | C16H12O5 | 284 | 267.98[M-H-CH_3_]^-^ ;270.05[ M+H-CH_3_]^+^;253.08[M+H-C H_4_O]^+^;  225.03[M+H-C_2_H_4_O_2_]^+^ | 94.0/94.0 | 97.5/97.5 | Ⅳ |

| 15 | 37.94 | 2-Hydroxy-3-methoxyst rychnine | 379.17 | 0.11 | — | — | C22H24N2  O_4_ | 380 | 297.16[M-H-C_5_H_8_N]^-^ | 13.0 | 78.0 | Ⅳ |
| --- | --- | --- | --- | --- | --- | --- | --- | --- | --- | --- | --- | --- |
| 16 | 38.43 | Rhamnocitin/ isomeride | 299.06 | 2.17 | — | — | C16H12O6 | 300 | 283.94[M-H-CH_3_]^-^ | 18.0/18.0 | 99.2/99.2 | Ⅳ |
| 17 | 43.57 | Prunetin/ isomeride | 283.06 | 2.76 | 285.07 | 2.81 | C16H12O5 | 284 | 267.93[M-H-CH_3_]^-^;  270.13[M+H-CH_3_]^+^;  253.03[M+H-CH_4_O]^+^ | 94.0/94.0 | 97.5/97.5 | Ⅳ |
| 18 | 45.81 | Methylnissolin | 299.09 | 1.44 | 301.11 | 3.22 | C17H16O5 | 300 | 284.04[M-H-CH_3_]^-^ ;  269.05[M+H-CH_4_O]^+^ | 224.0 | 69.4 | Ⅳ |
| 19 | 49.48 | Calycosin* | 283.06 | -0.35 | 285.08 | 2.28 | C16H12O5 | 284 | 268.05[M-H-CH_3_]^-^;  270.02[M+H-CH_3_]^+^;  253.06[M+H+CH_4_O]^+^ | 106.0 | 96.5 | Ⅳ |
| 20 | 55.42 | Formononetin* | 267.07 | 1.70 | — | — | C16H12O4 | 268 | 251.95[M-H-CH_3_]^-^ | 191.0 | 87.2 | Ⅳ |
| 21 | 9.11 | 1,2-benzenedicarboxyli c acid, bis(2-methyl-  propyl) ester | 277.09 | 2.00 | — | — | C11H18O8 | 278 | 114.90[M-H-C_6_H_10_O_5_]^-^ | 244.0 | 97.2 | Ⅴ |
| 22 | 17.47 | 1, 2-  benzenedicarboxylic acid,bis(2-methylpropyl  ) ester | 277.13 | 2.96 | 279.14 | 7.96 | C15H20O4  N | 278 | 235.01[M-H-C_2_H_2_O]^-^ ;  219.08[M+H-C_2_H_4_O_2_]^+^;  262.13[M+H-OH]^+^;  200.97[M+H-C_6_H_7_]^+^; | 224.0 | 99.8 | Ⅴ |
| 23 | 21.75 | Dimethyl phthalate | 193.05 | 4.38 | — | — | C10H10O4 | 194 | 148.97[M-H-C_2_H_5_O]^-^; | 220.0 | 99.9 | Ⅴ |
| 24 | 28.09 | Cinnamic acid  isomeride | 147.05 | 7.07 | — | — | C_9_H_8_O_2_ | 148 | 102.92[M-H-CO_2_]^-^ | 27.0 | 99.1 | Ⅴ |
| 25 | 28.86 | 2,3,5-  trimethoxytoluene | 181.09 | 5.47 | — | — | C10H14O3 | 182 | 121.93[M-H-C_3_H_7_O]^-^  136.90[M-H-CO_2_]^-^ | 220.0 | 99.6 | Ⅴ |
| 26 | 29.5 | Isobutyl benzoate | 223.10 | 2.98 | — | — | C11H14O2 | 224 | 208.04[M+CHO_2_-CH_3_]^-^;  178.97[M+CHO_2_-CO_2_]^-^ | 245.0 | 99.8 | Ⅴ |
| 27 | 33.73 | (+)-syringaresinol | 417.16 | 1.45 | — | — | C22H26O8 | 418 | 180.92[M-H-C_12_H_12_O_5_]^-^；  402.20[M+H-CH_3_]^-^ | 77.0 | 99.2 | Ⅴ |
| 28 | 36.75 | Dimethyl phthalate | 193.05 | 5.36 | — | — | C10H10O4 | 194 | 148.94[M-H-CO_2_]^-^;  177.99[M-H-CH_3_]^-^;  133.98[M-H-C_3_H_7_O]^-^ | 220.0 | 99.9 | Ⅴ |
| 29 | 39.24 | (+)-syringaresinol | 417.16 | 1.45 | — | — | C22H26O8 | 418 | 181.01[M-H-C_14_H_20_O_3_]^-^;  402.17[M-H-CH_3_]^-^ | 77.0 | 99.2 | Ⅴ |
| 30 | 42.31 | Llicoflavonol  /Isolicoflavonol | — | — | 355.17 | 2.38 | C16H28O7 | 354 | 266.98[M+Na-C_4_H_24_O]^+^;  285.01[M+Na-C_3_H_18_O]^+^ | 21.0/21.0 | 85.6/85.6 | Ⅴ |

| 31 | 49.47 | Cinnamic acid * | 147.05 | 7.44 | — | — | C_9_H_8_O_2_ | 148 | 102.92[M-H-CO_2_]^-^ | 27.0 | 99.1 | Ⅴ |
| --- | --- | --- | --- | --- | --- | --- | --- | --- | --- | --- | --- | --- |
| 32 | 57.61 | Trans-2-Nonenal | 139.11 | 7.90 | — | — | C_9_H_16_O | 140 | 70.72[M+H-C_5_H_8_]^-^;  110.93[M+H-CO]^-^ | 243.0 | 99.7 | Ⅴ |
| 33 | 16.75 | Talatizidine | — | — | 408.27 | 3.79 | C23H37O5  N | 407 | — | 20.0 | 87.7 | Ⅵ |
| 31 | 22.7 | Talalisamine | — | — | 422.29 | 3.63 | C_24_H_39_NO  5 | 421 | — | 20.0 | 87.7 | Ⅵ |
| 35 | 33.11 | 14-benzoyl-10-OH-mes aconine | 650.28 | 1.95 | 606.29 | 2.62 | C31H43O11 N | 605 | 633.25[M+CHO_2_-OH]^-^ ;  556.39[M+H-C_2_H_2_N];  524.41[M+H-C_5_H_8_N];  588.48[M+H-H_2_O]^+^ | 8.0 | 96.4 | Ⅵ |
| 36 | 36.04 | 14-benzoyl-10-OH-aco nine | 664.30 | 2.83 | 620.30 | 6.05 | C32H45O11 N | 619 | 633.11[M+CO_2_H-CH_3_O]^-^  570.43[M+H-C_4_H_2_]^+^ | 7.0 | 95.5 | Ⅵ |
| 37 | 38.89 | Benzoylmesaconine* | 634.29 | 0.82 | 590.29 | 5.39 | C_31_H_43_NO  10 | 589 | 616.38[M+CHO_2_-H_2_O]^-^;  588.45[M+CHO_2_-CH_2_O_2_]^-^  540.43[M+H-C_4_H_2_];  558.43[M+H-CH_4_O]^+^ | 13.0 | 98.3 | Ⅵ |
| 38 | 40.32 | Benzoylaconitine* | — | — | 604.31 | 4.11 | C_32_H_45_NO  10 | 603 | 554.27[M+H-C_4_H_2_]^+^;  572.45[M+H-CH_4_O]^+^ | 22.0 | 99.3 | Ⅵ |
| 39 | 41.92 | Benzoylhypacoitine* | 618.29 | 0.004 | 574.30 | 4.53 | C_31_H_43_NO  9 | 573 | 586.17[M+CHO_2_-CH_4_O]^-^ ;  524.41[M+H-C_4_H_2_]^+^;  542.42[M+H-CH_4_O]^+^ | 78.0 | 99.7 | Ⅵ |
| 40 | 42.76 | Benzoyldeoxyaconine | 632.31 | 4.11 | 588.31 | 3.52 | C32H45O9  N | 587 | 614.32[M+CHO_2_-H_2_O]^-^ ;  556.44[M+H-CH_4_O]^+^ | 19.0 | 98.6 | Ⅵ |
| 41 | 43.52 | Beiwutine/10-OH-mesa  conitine | — | — | 648.30 | 2.44 | C_33_H_45_NO  12 | 647 | 588.47[M+H-C_2_H_4_O_2_]^+^ | 9.0/14.0 | 96.4/98.5 | Ⅵ |
| 42 | 46.28 | Hypaconitine* | — | — | 616.31 | 4.62 | C_33_H_45_NO  10 | 615 | 556.40[M+H-C_2_H_4_O_2_]+;  524.46[M+H-C_7_H_8_]^+^ | 1250 | 99.7 | Ⅵ |
| 43 | 46.28 | Aconitine * | — | — | 646.32 | 2.91 | C34H47NO1  1 | 645 | 566.43896[M+H-C_5_H_5_O]^+^ | 45.0 | 99.6 | Ⅵ |

Ⅱ：Raidix Paeoniae Alba (Bai Shao), Ⅲ：Radix Glycytthizae (Gan Cao),Ⅳ:R adix Astragali (Huang Qi), Ⅴ：Herba Ephedrae (Ma Huang), Ⅵ：Radix Aconiti (Wu Tou), *：Being identified with Standard substance, chemical constituents in red have good intestinal absorption, chemical constituents in blue have good oral bioavailability.

**Table S3. Pharmacokinetics parameters of Paeoniflorin and Liquiritin in the plasma of the rats received the two-BAC-combination and WTD respectively via pharmacokinetic analysis (non-compartment analysis, n=3)**

**PK parameters***

|  | **2-BAC** | **WTD** | **2-BAC** | **WTD** |
| --- | --- | --- | --- | --- |
| *C*_max_ (ng/mL) | 668 | 144 | 17.1 | 13.3 |
| *T*_max_ (h) | 0.08 | 2.00 | 0.08 | 0.50 |
| AUC_0-t_(ng∙h/mL) | 1047 | 720 | 44.1 | 50.0 |
| AUC_0-∞_(ng∙h/mL) | 1061 | -- | 45.7 | -- |
| *t1/2_lamda_Z* (h) | 1.02 | -- | 1.37 | -- |
| MRT (h) | 1.35 | -- | 1.83 | -- |

**Paeoniflorin Liquiritin**

***** The values were expressed as mean ± standard deviation. *C*_max_, measured maximum concentration; *T*_max_, measured peak time; *t*_1/2_, half life time; AUC_0–t_, area under curve from time zero to the time of last quantifiable concentration; AUC_0–∞_, area under curve from time zero to infinity; MRT, mean residence time.

# Table S4 List of neuropathic pain (NP)-related genes collected from Drugbank and OMIM databases

| **Databases** | **NP-related genes** |
| --- | --- |
| Drugbank | A2M, ABAT, ABCC1, ABCC2, ABL1, ACHE, ADH1A,ADH1B, ADH1C, ADORA1, ADORA2A, ADRA1A, ADRA1B, ADRA1D, ADRA2A, ADRA2B, ADRA2C, ADRB1, ADRB2, ADRB3, AHR, AKR1C1, ALOX5, ANPEP, AOC3, AR, ASIC1, ASIC3, ATP4A,ATP6V1A, AVPR1A, AVPR1B, AVPR2, B4GALT1, B4GALT2, B4GALT3, B4GALT4, BCAT1, BCHE, BCL2, BCR/ABLFUSIO, BDKRB2, BLAZ, BRAF, C1QBP, C5, CA1, CACNA1A, CACNA1B, CACNA1C, CACNA2D1, CACNA2D2, CALCA, CALCB, CALCR, CAMC, CARTPT, CCL2, CCL3, CD44, CFTR, CHRM1, CHRM2, CHRM3, CHRM4, CHRM5, CHRNA1, CHRNA10, CHRNA2,CHRNA3, CHRNA4, CHRNA7, CHRNA9, CHRNB2, CHRNB4, CLCN2, CLCNKA, CNR1, CNR2, COBT, COMT, CRBN, CSF1R, CTLA4, CXCR1, CYP11B1, CYP17A1, CYP1A2, CYP27B1, CYSLTR1, DACA, DACB, DACC, DDR1, DDR2, DPP4, DRD1,DRD2,DRD3,DRD4,DRD5,E,EDNRA,EGFR,ELN, EPHA2, ESR1, ESR2, ESRRG, FAAH, FABP2, FCGR1A, FDPS, FGA, FGF1, FGF2, FGF4, FGFR1, FGFR2, FLT1, FLT4, FN1, FPGS, FRK, FTSI, GABBR1, GABBR2, GABRA1, GABRA2, GABRA3, GABRA4, GABRA5, GABRA6, GABRB1, GABRB2, GABRB3, GABRD, GABRE, GABRG1, GABRG3, GABRP, GABRQ, GABRR1, GABRR2, GABRR3, GAD65, GDNF, GGPS1, GLO1, GLP2R, GLRA1, GLRA2, GLRA3, GNRHR, GNRHR2, GPT, GPT2, GRIA2, GRIK2, GRIN1, GRIN2A, GRIN2B, GRIN2C, GRIN2D,  GRIN3A, GRIN3B, GSTP1, GUCY2C, HABP2,HABP4, |

|  | HAPLN1, HAPLN3, HAPLN4, HDAC2, HLA-DRB1, HMGCR, HMMR, HRH1, HRH2, HRH3, HRH4, HSPA5,HTR1A, HTR1B, HTR1D, HTR1E, HTR1F, HTR2A, HTR2B, HTR2C, HTR3A, HTR6, HTR7, ICAM1, IFNG, IKBKB, IL10, IL1B, IL1R1, IL5, IL6, IMPDH1, IMPDH2, IMPG2, JAK1, JAK2, JAK3, KCNA1, KCND2, KCND3, KCNH2, KCNJ1, KCNJ11, KCNJ8, KCNMA1, KCNQ1, KCNQ2, KCNQ3, KCNQ4, KCNQ5, KDR, KEAP1, KIT, LAYN, LHCGR, LTF, MAOA, MAOB, MAPK11, MAPK14, MPL, MRCA, MRCB, MRDA, MT-CO2, MT-ND5, MUC2, N/A, NAGK, NAGLU, NAGPA, NCAN, NFKB1, NFKB2, NFKBIA, NGF, NISCH, NOS1, NOS2, NPRS, NPY2R, NR3C1, NTF3, NTF4, NTRK1, NTRK2, OPRD1, OPRK1, OPRL1, OPRM1, ORM1, ORM2, PAGA,PBP1B, PBP2A, PBP3, PBPA, PCCB, PDE3A, PDE4A, PDE5A, PDE6G, PDE6H, PDGFRA, PDGFRB, PDPK1, PENA, PGF, PGR, PGRMC1, PKN3, PLA2G1B, PLA2G2A, PLA2G2E, PLA2G4A, PLAT, POL, POLB, POMC, PONB, POR, PPARA, PPARG, PRKAA1, PRKAA2,PRKAB1, PRKAB2, PRKAG2, PRKAG3, PTGDR2, PTGER1, PTGIS, PTGR2, PTGS1, PTGS2, PTH1R, PTPRS, RAF1, RENBP, RET, RHOB, RNASE3, RPS6KA3, RXRA, SCN10A, SCN1A, SCN2A, SCN3A, SCN4A, SCN5A, SCN8A, SCN9A, SERPINF2, SI, SIGMAR1, SLC18A2, SLC25A4, SLC25A5, SLC25A6, SLC6A2, SLC6A3, SLC6A4, SMPD1, SNAP25, SNF, SST, SSTR1, SSTR2, SSTR3, SSTR5, STAB2, SYT2, TAAR1,TACR1, TACR3, TEK, THBD, TK, TLR2, TLR4, TLR7, TLR8, TLR9, TNF, TNFAIP6, TNFSF11, TNFSF13B, TNNC1, TP53, TPMT, TPO,TRDMT1, TRIM13, TRPA1,  TRPM8, TRPS, TRPV3, TSPO, TUBB, TUBB1,TYMS, |
| --- | --- |

|  | UGT1A9, UL30, VAMP1, VAMP2, VARS, VCAN, VDAC1,  VDAC2, VDAC3, VDR, VEGFA, VEGFB, WARS, WARS2 |
| --- | --- |
| OMIM | AAS, ABCG5, ACADVLD, ACCN1, ACCN2, ACCN3,ACTC1, ACTG2, ADCAD1, ADCY1, ADCY8, ADHR, ADM, ADORA2A, AGER, AGS5, AKT1, AKU, ALAD, ALDD, ALDOB, ALMS, ALPL, ALS21, AN, ANKH, APOA5, APOC2, APOE, AR, ARH, ARHR1, ARRB2, ARSA, ARTN, ARVD5, ATL1, ATP2A1, ATS, B2M, BCNS, BCPM, BDCHS,BDKRB1, BDKRB2, BDMR, BDNF, BMD, BOS, BRGDA5, BRRS, BTG2, BTK, BWS, CACNA1A, CACNA1B, CACNA1G, CACNA2D1, CACNA2D2, CACNA2D3, CACNA2D4, CACNB3, CACNG2, CACP, CALJA, CAMK4, CARASIL, CAV3,CBS, CCAL2, CCR2, CCR5, CCT4, CD, CD209, CD40LG, CDB2, CDG1O, CDK5, CDK5R1, CDSP, CECR1, CF, CFTD, CHRNA10, CHRNA9, CINCA, CIP, CIPA, CISS1, CISS2, CLCN1, CLCN6, CLCNKB, CMD1A, CMD1S, CMD1Y, CMD3B, CMH1, CMH18, CMH4, CMT1A, CMT1B, CMT2A2, CMT2B, CMT2F, CMT2O, CMT2P, CMT4B3, CMT4J, CMTRIA, CMTRIB, CNNM4, CNR1, CNR2, CNTN1, COL11A2, COL1A1, COL1A2, COL2A1, COL3A1, COL5A2, COL6A1, COL9A2, COL9A3, COMP, COMT, COX20, COX6B1, CPS1, CPT2, CRLF1, CSF2, CSF2RA, CSF3, CSF3R, CSNK1D, CSS, CTS1, CTSC, CVS, CWS1, CYP19A1, CYP2D6, DAR, DDH1, DDH2, DIAR6, DIAR7, DJS,DLD,DLDD,DM1,DM2,DMD,DPYSL2,DRGX,DRP2, DSMA1, DUH3, DYNC1H1, DYT16, EA1, EDM2, EDM3, EDM5, EDM6, EDMD1, EDN1, EDNRB, EGR2, EIEE11, EIF2AK3, ELANE, EMD, EOE1, ERBB4, ESCO2,ESTRR,  F12, F2RL1, F7, FAAH, FASPS2, FBN1, FBN2, FCAS1, |

|  | FCAS2, FEO, FEPS1, FEPS2, FEPS3, FGA, FHM1, FILS, FMF, FOP, FRTS2, FSHD2, FSHR, FTL, FXN, FXTAS, G6PD, GABBR1, GABRA2, GABRA3, GAD2, GAL, GALR2, GALR3, GBA, GBD1, GBS, GCH1, GDNF, GHDD, GHSR, GLA, GLB1, GLRA3, GNAI2, GNAO1, GNE, GNPTAB, GNPTAG, GNPTG, GPIHBP1, GPR74, GRP, GRPR, GSD, GSD10, GSD11,GSD2, GSD3, GSD5, GSD7, GSD9D, GSTA1, HADHA, HADHB, HAE1, HAE3, HBB, HBG2, HCN2, HCP,HEMA, HEXB, HFE1, HFE2A, HFE4, HFTC, HHT, HIDS, HJCYS, HLRCC, HMAG, HMBS, HMN2D, HMSNR, HNA, HNF1A, HOMER1, HOMG3, HPGD, HRPT2, HSA, HSAN1A, HSAN1C, HSAN2A, HSAN2B, HSAN3, HSAN5, HSAN6, HSAN7, HSN1D, HSN1E, HSN1F, HSN2C, HTR1B, HTR1E, HTR2A, HTR3A, HYPP, IBD1, IBMPFD1, ICP3, IDD, IFNGR1, IL1B, IL33, JBS, JPS, KCNIP3, KCNJ1, KCNJ2, KIF1A, KIT, KNG1, KRAS, L1CAM, LAM, LBR, LDS2, LDS3, LEP, LGMD2H, LGMD2L, LGMD2S, LIFR, LPIN1, LPL, LRP1, LYZ, MADA, MADD, MAPT, MARS, MAS, MAS1L, MBS, MC1R, MCKD1, MCM6, MDDGC5, MDDGC7,MECP2, MEFV, MEN1, MEN2A, MEN2B, MFM1, MFM5, MFN2, MFS, MGR13, MGS, MJD, MLYCD, MMDD, MMP2, MMP9,MNG1, MNS, MODY8, MOPD2, MOWS, MPD4, MPO, MPS9, MRD18, MRD24, MRGPRD, MRGPRE, MRGPRF, MRGPRG, MRGPRX1, MRGPRX2, MRGPRX3, MRGPRX4, MRMV1, MRMV2, MTCYB, MTDPS1, MTDPS12,MTDPS4A, MTDPS4B, MTDPS6, MTTK, MVCD1, MVP, MWS, MYH7, MYOT, NAV2, NF1, NF2, NFKB1, NGF, NLRP12, NLSDM, NPS, NPY, NPY1R, NR0B1, NS1, NS4, NTRK1, OD,OFD1,  OI13, OMPP, OPA1, OPLAHD, OPRD1, OPRK1,OPRM1, |
| --- | --- |

|  | OPTA1,OPTA2, OPTB6, OS1, OS3, OSCS, OSMED, P2RX3, P2RX4, P2RX5, PAN, PARK6, PCLD, PCTT, PDB, PENK, PEOA3, PEOA4, PEPS, PFKM, PGAM2, PGL1, PGL4, PHOAR1, PJS, PKD1, PKD2, PKHD1, PLOD3, PLOSL, PMC, PMPCB, PNH1, PNH2, PNMHH, PNOC, PNPLA6, POMK, PPAC, PPKNEFD, PPOX, PRKCE, PRKCZ, PRLHR, PROK2, PROL1, PRX, PSACH, PTGER1, PTGER2, PTGES, PTGIR, PTGS1, PTGS2, PWS, PYGM, RAD54L, RCDP1, RHUC1, RLN2, RMD, RMD1, ROR2, RRS, RTPS2, RVCL, SCAR4, SCDO5, SCN1, SCN10A, SCN11A, SCN1B, SCN2A, SCN4A, SCN9A, SDHB, SHANK3, SIM2, SLC12A3, SLC16A1, SLC17A6, SLC17A8, SLC34A2, SMA3, SMARCB1, SMAX1, SMDP2, SMN1, SMPD1, SMS, SOS1, SOST, SOX9, SPDA1, SPG3A, SPG4, SPG7, SPG8, SPINK1, SPS, SPTLC1, SRXX2, SSX1, STL1, STL3, STOML3, STRMK, TAC1, TACR1, TDH3, TGD, TH, THPH3, TIMM8A, TLR4, TNFRSF11B, TNFRSF1A, TNNC1, TNXB, TREH, TRPA1, TRPC1, TRPM2, TRPM5, TRPM8, TRPS1, TRPV2, TRPV4, TTR, TYRSN1, UFS1, UROD, VANGL1, VDDR2A, VHL, VSCM, VUR4, WNK1,WVS,  XMPMA, YARS2, |
| --- | --- |

**Supplementary Figure legends**

**Figure S1. Molecular network-based investigation of material basis and underlying mechanisms of WTD acting on NP.** (a) Common targets of WTD and known FDA-approved analgesic agents. (b) Top 10 NP-related pathways enriched by the WTD putative targets. (c) The interaction network of "NP-related genes-WTD candidate targets" illustrated using the links of NP-related genes and WTD candidate targets. Rectangle nodes refer to the NP-related pathways enriched by WTD candidate targets. Circle nodes refer to the WTD candidate targets. Circle nodes with red highlights refer to the common targets of WTD and known FDA-approved analgesic agents. (d) The schematic diagram of the underlying mechanisms of WTD against NP via reversing the neuroinflammation by targeting the *CCL5-CCR5-GNAI1-SRC-PIK3CA-AKT* signal axis.

**Figure S2. The chemical structure of PAE and LIQ, and molecular docking simulation of the binding pattern of them with corresponding proteins with strong binding efficiency.** (a) and (b) The structures of PAE and LIQ prepared by ChemDraw. The molecular docking simulation of the binding pattern of PAE with CCL5 (c), CCR5 (d), GNAI1 (e), SRC (f), PIK3CA (g), AKT (h). The molecular docking simulation of the binding pattern of LIQ withCCL5 (i), CCR5 (j), GNAI1 (k), SRC (l), PIK3CA (m), AKT (n). The docking scores indicate the binding energy between the ligand and the receptor, and may be in units of kcal/mol. The higher absolute value of docking score represents stronger binding efficiency.

**Figure S3. The binding affinity of PAE or LIQ with CCL5, and the concentration of PAE and LIQ in plasma after the single treatment of the two-BAC combination or WTD respectively.** (a) The surface plasmon resonance (SPR) assay of the interaction of PAE with CCL5 protein for different concentrations of compounds (RU, resonance unit). (b) The SPR assay of the interaction of LIQ with CCL5 protein for different concentrations of compounds(RU, resonance unit).All binding affinity values are determined according to three parallel experiments. (c)~(d) The mean plasma concentration-time curves of PAE and LIQ after the single treatment of the two-BAC combination or WTD (n=3).

**Figure S4. Characteristics of anti-hyperalgesia of the two-BAC combination on SNL and MIP-induced NP.** SNL induced mechanical allodynia and cold hyperalgesia of rats successfully. The two-BAC combination significantly reversed SNL induced mechanical allodynia (a) and cold hyperalgesia (b); MIP led to obvious mechanical allodynia (c) and cold hyperalgesia (d), the anti-hyperalgesia effects of the two-BAC combination was similar with WTD. Data are represented as mean±s. ^###^, significantly different from Sham group (P<0.001); ^*^, ^**^, and ^***^, significantly different from SNL group (P<0.05, 0.01, and 0.001, respectively, n=5).

**Figure S5. The influence of the two-BAC combination on GFAP, NeuN and CCL5 in the dorsal horn of L5 spinal cord tissues of SNL rats.** (a) The representative photographs of the immunostainings of GFAP, NeuN and CCL5 proteins in different groups. (b)~(d) The evaluation of the immunoreactive scores of GFAP, NeuN and CCL5 proteins in different groups. Data are represented as mean±s. ^#^ and ^##^, significantly different from Sham group (P<0.05, and 0.01, respectively); ^*^ and ^**^, significantly different from SNL group (P<0.05, and 0.01, respectively, n=3).

**Figure S6. The indexes of the heart (a), liver(b), kidney(c), lung(d) and spleen(e) of rats in different groups.** Data are represented as mean±s. ^###^, significantly different from Sham group (P<0.001); ^*^, ^**^, and ^***^, significantly different from SNL group (P<0.05, 0.01, and 0.001, respectively, n=6).

**Figure S7. The mRNA expression levels of CCL5 (a), CCR5 (b), GNAI1 (c), SRC (d), PIK3CA (e) and AKT (f) in the dorsal horn of L5 spinal cord tissues in different groups.** Data are represented as mean ± s. ^#^, ^##^, ^###^, significantly different from Sham group (P<0.05, 0.01, and 0.001, respectively) ; ^*^, ^**^, and ^***^, significantly different from SNL group (P<0.05, 0.01, and 0.001,respectively); ^&^, ^&&^, ^&&&^, significantly different from the two-BAC combination group (P<0.05, 0.01, and 0.001, respectively, n=3).

**Figure S8**.**The protein expression levels of CCL5 (a), CCR5 (b), GNAI1 (c), p-SRC (d), p-PIK3CA (e) and p-AKT (f) in the dorsal horn of L5 spinal cord tissues in different groups, as well as the serum levels of TNF-α (g), IL-1β (h), and IL-6 (i) in different groups.** Data are represented as mean ± s. ^#^, ^##^, ^###^, significantly different from Sham group (P<0.05, 0.01, and 0.001,respectively) ; *, ^**^, and ^***^, significantly different from SNL group (P<0.05, 0.01, and 0.001, respectively); ^&^, ^&&^, ^&&&^, significantly different from the two-BAC combination group (P<0.05, 0.01, and 0.001,respectively, n=3).
