# Supplementary figures and images for: Biomolecular network-based synergistic drug combination discovery: a combination of paeoniflorin and liquiritin alleviates neuropathic pain by inhibiting neuroinflammation via suppressing the chemokine signaling pathway
Source: Signal Transduct Target Ther. 2020 May 22;5:73. doi: 10.1038/s41392-020-0160-8 (PMC7242454; doi:10.1038/s41392-020-0160-8)

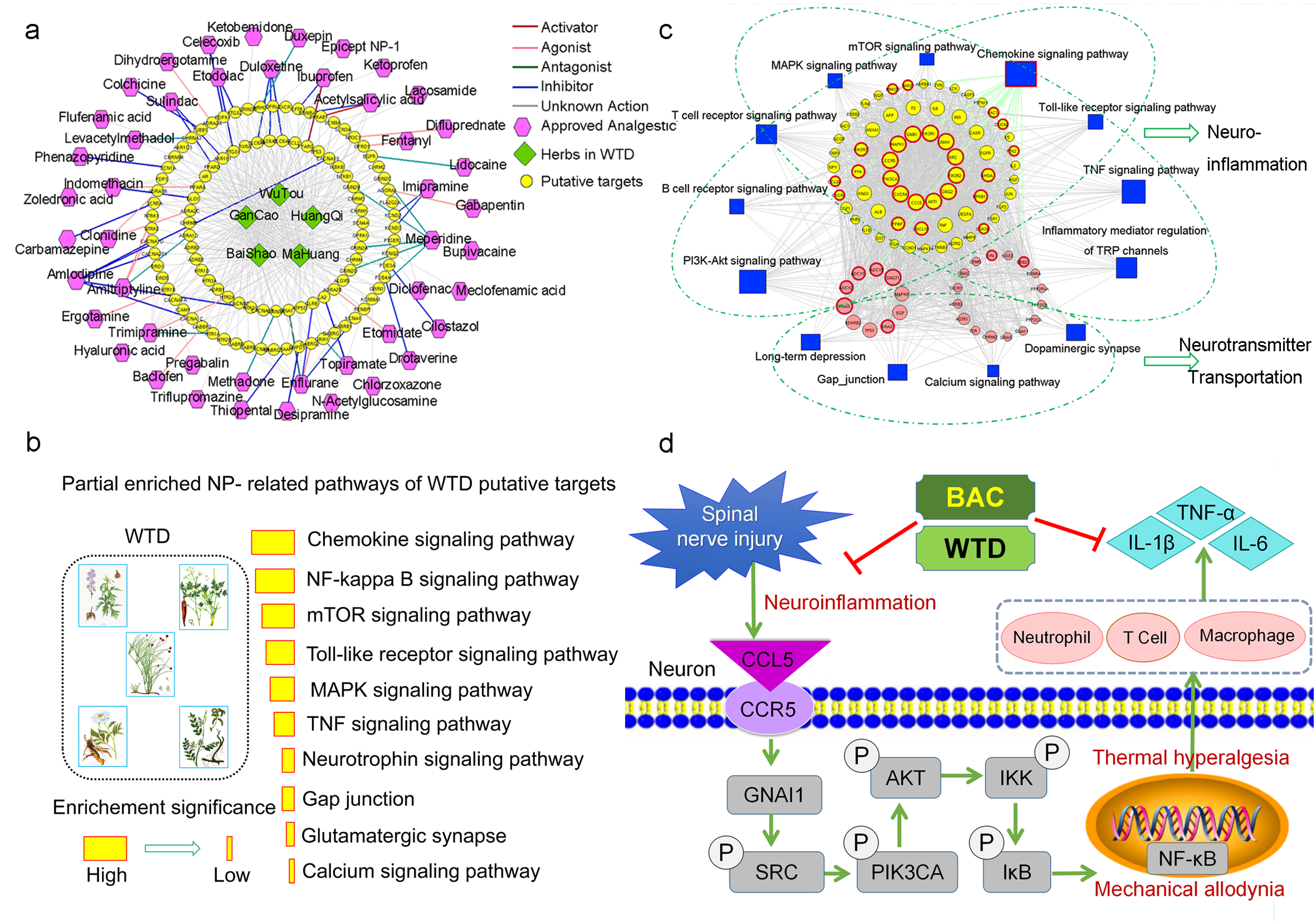

Supplement: Supplementary file 2 — Fig.S1 [file 41392_2020_160_MOESM2_ESM.tif]

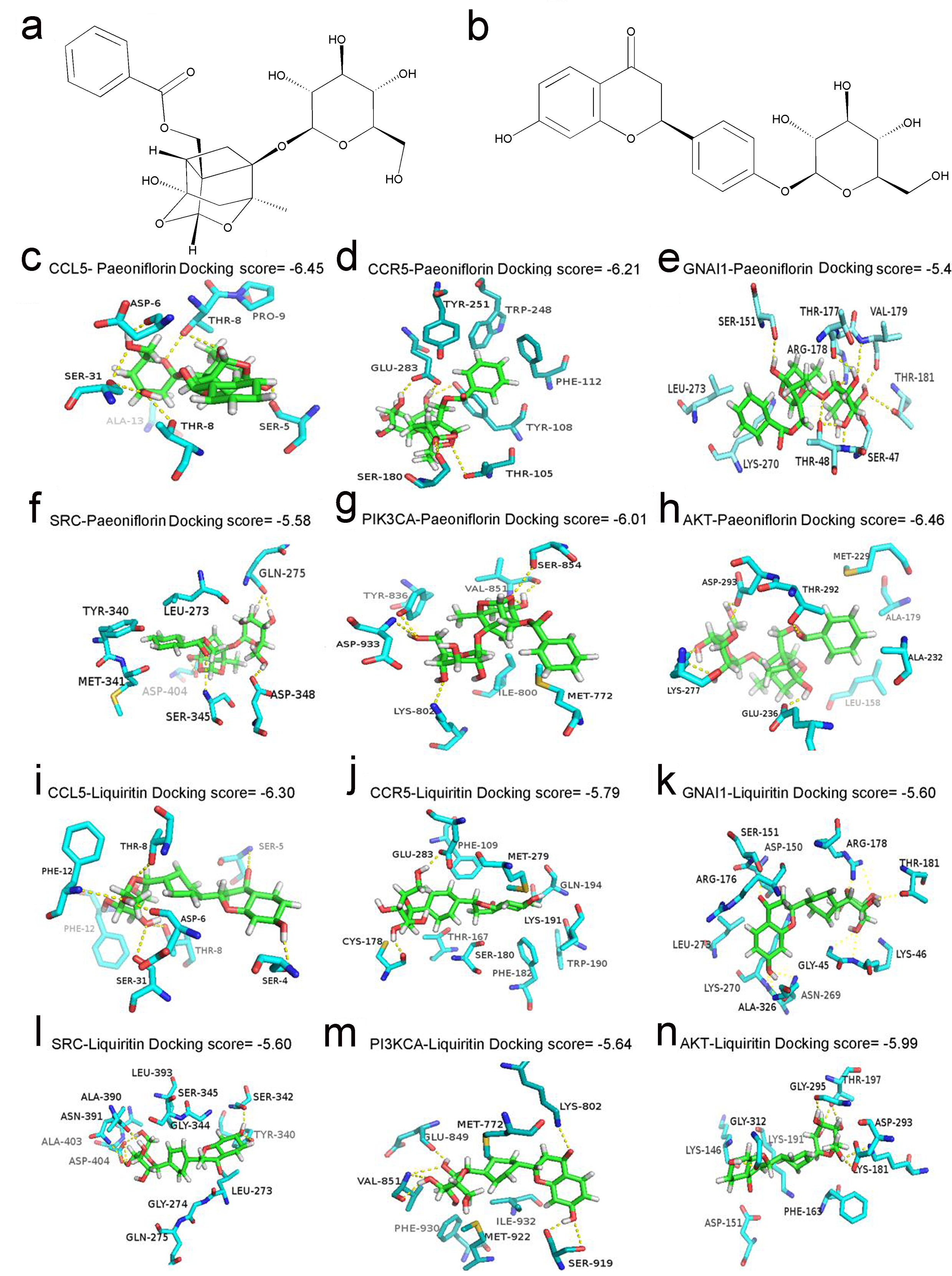

Supplement: Supplementary file 3 — Fig.S2 [file 41392_2020_160_MOESM3_ESM.tif]

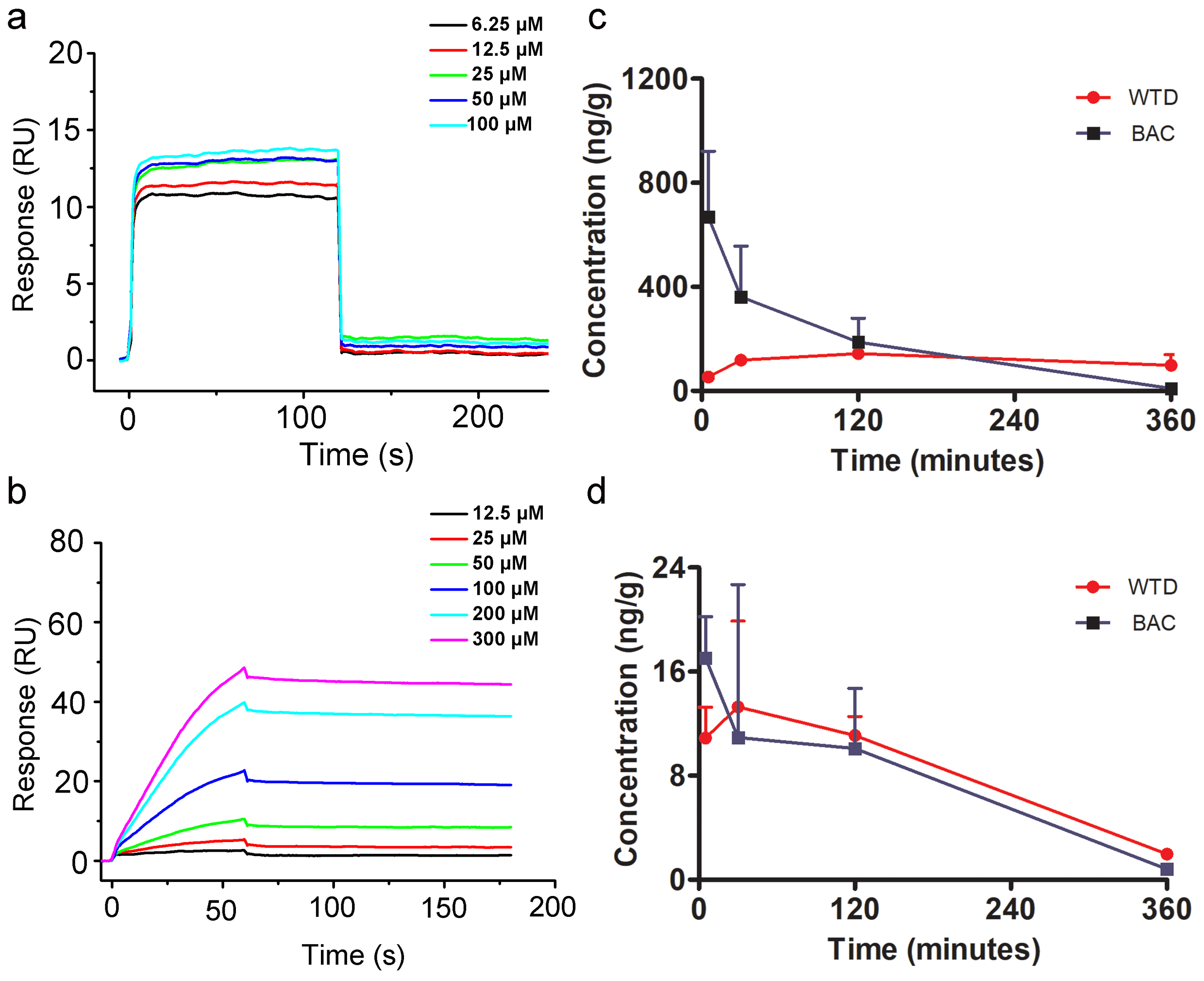

Supplement: Supplementary file 4 — Fig.S3 [file 41392_2020_160_MOESM4_ESM.tif]

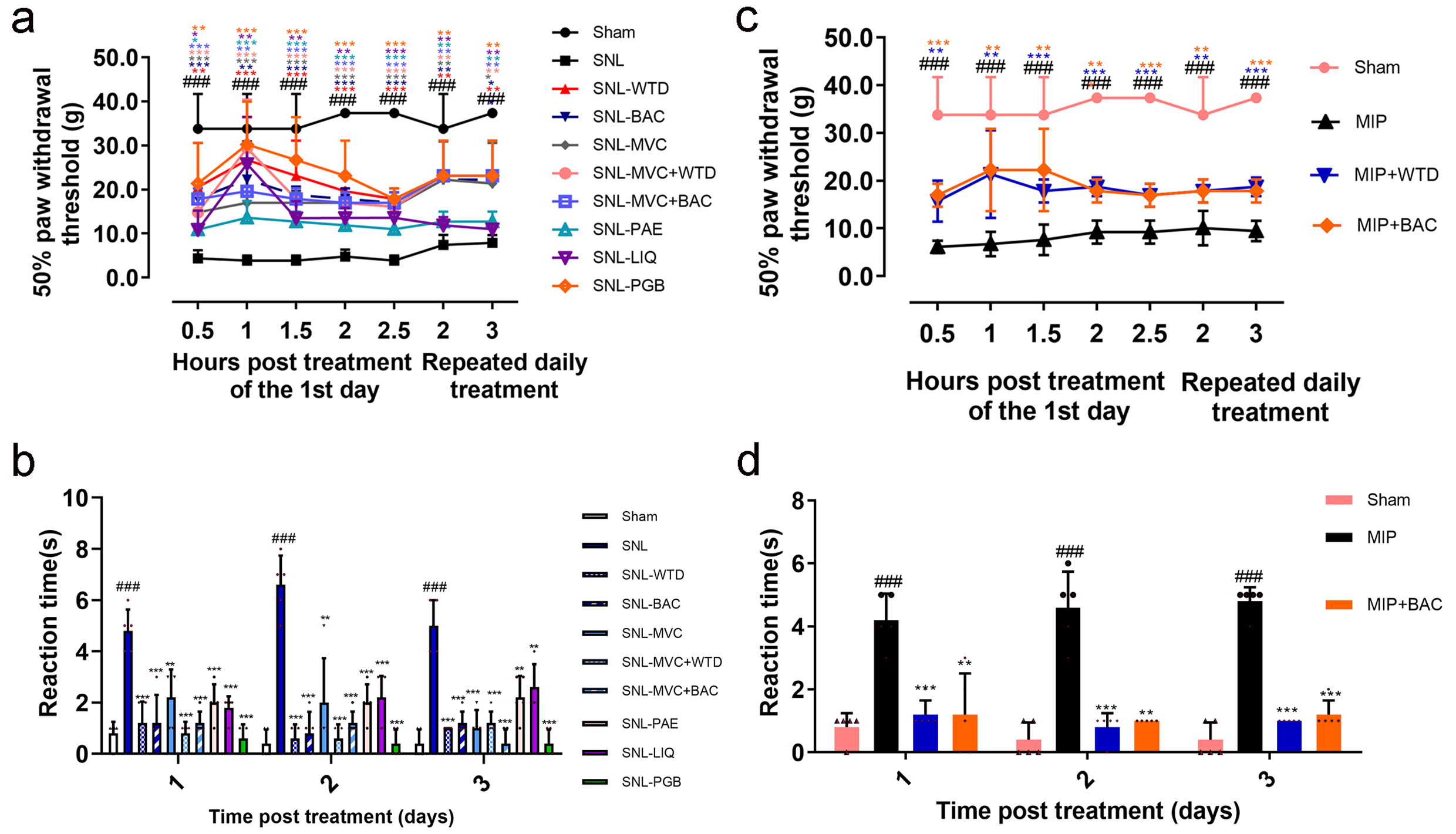

Supplement: Supplementary file 5 — Fig.S4 [file 41392_2020_160_MOESM5_ESM.tif]

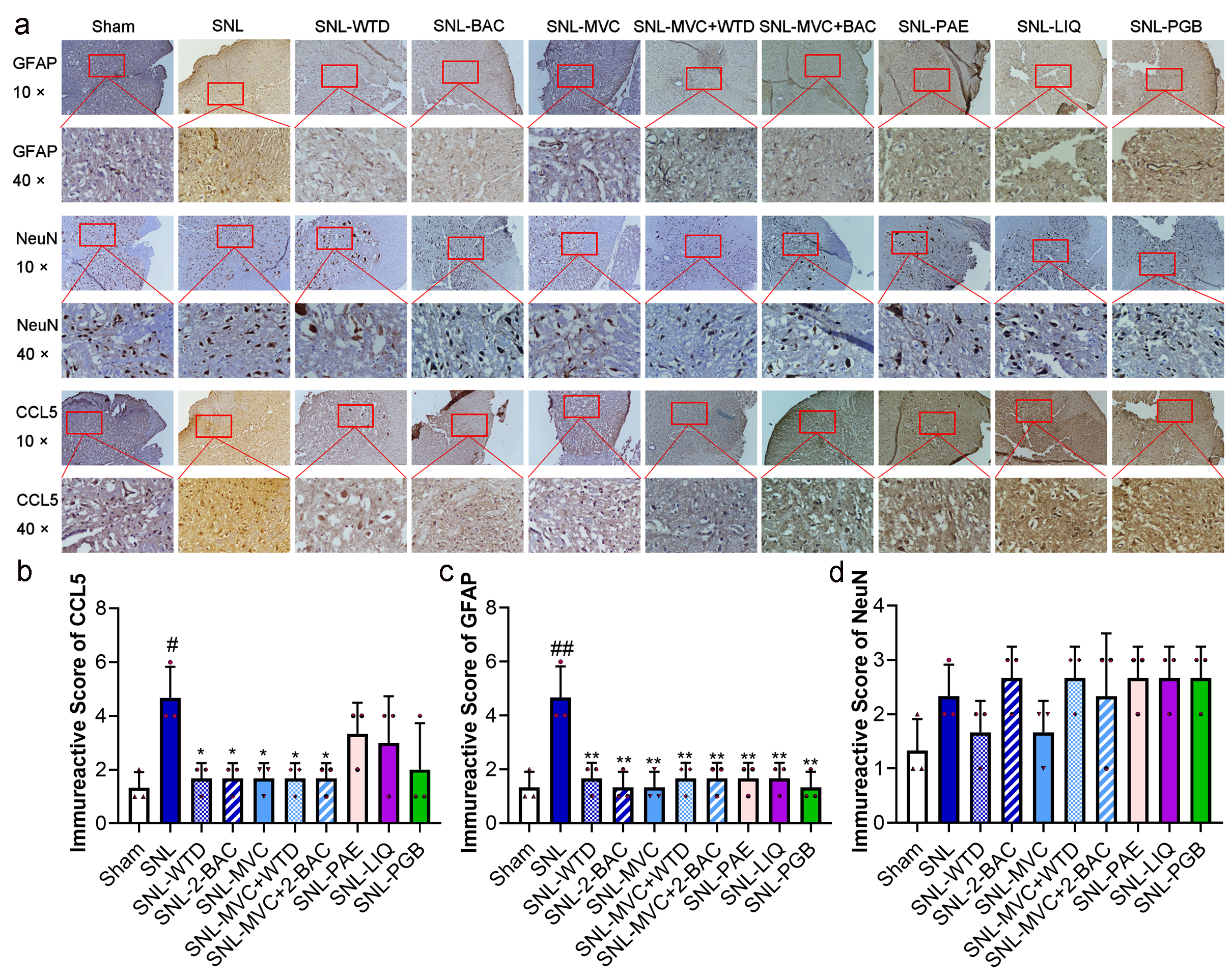

Supplement: Supplementary file 6 — Fig.S5 [file 41392_2020_160_MOESM6_ESM.tif]

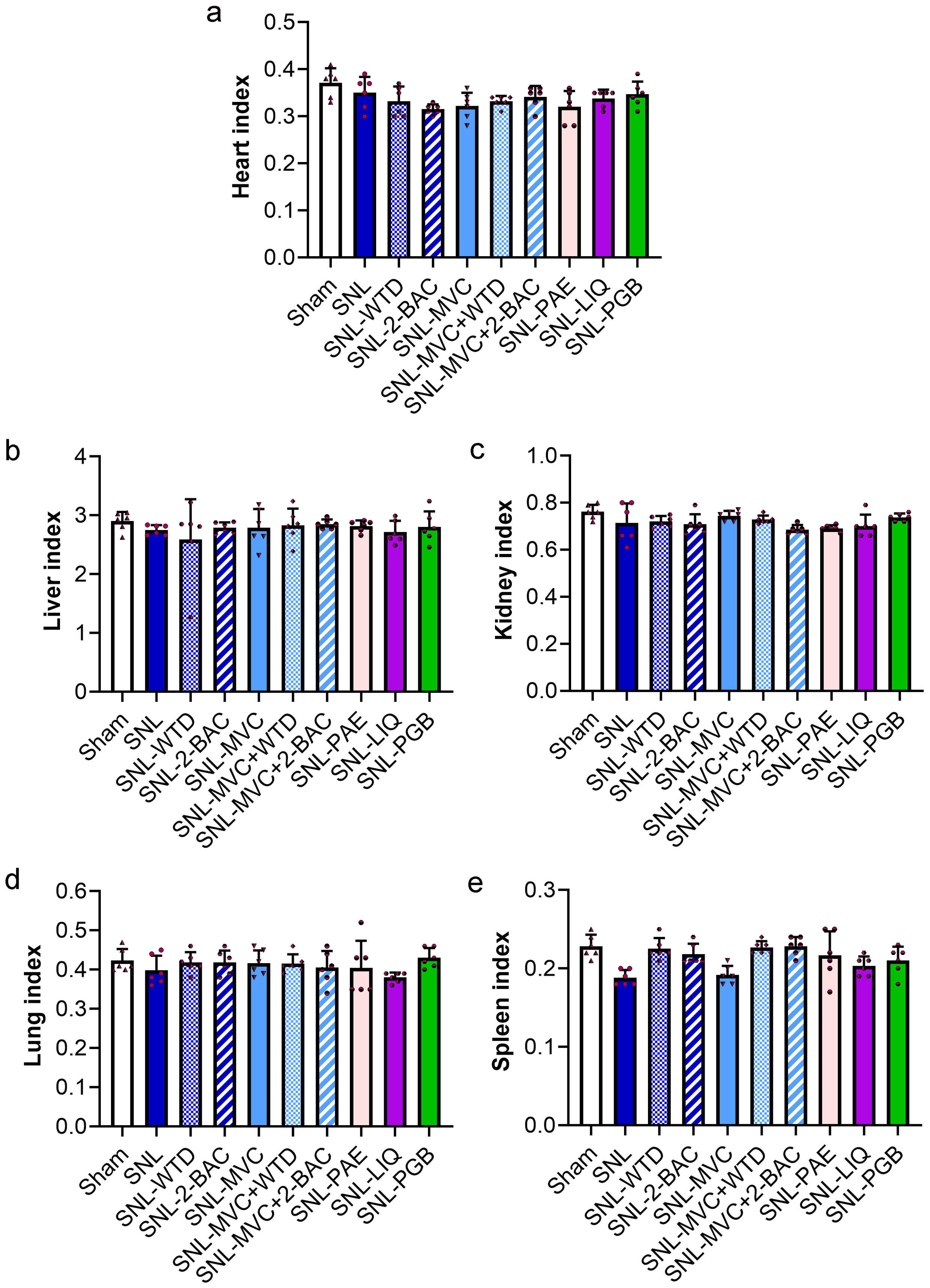

Supplement: Supplementary file 7 — Fig.S6 [file 41392_2020_160_MOESM7_ESM.tif]

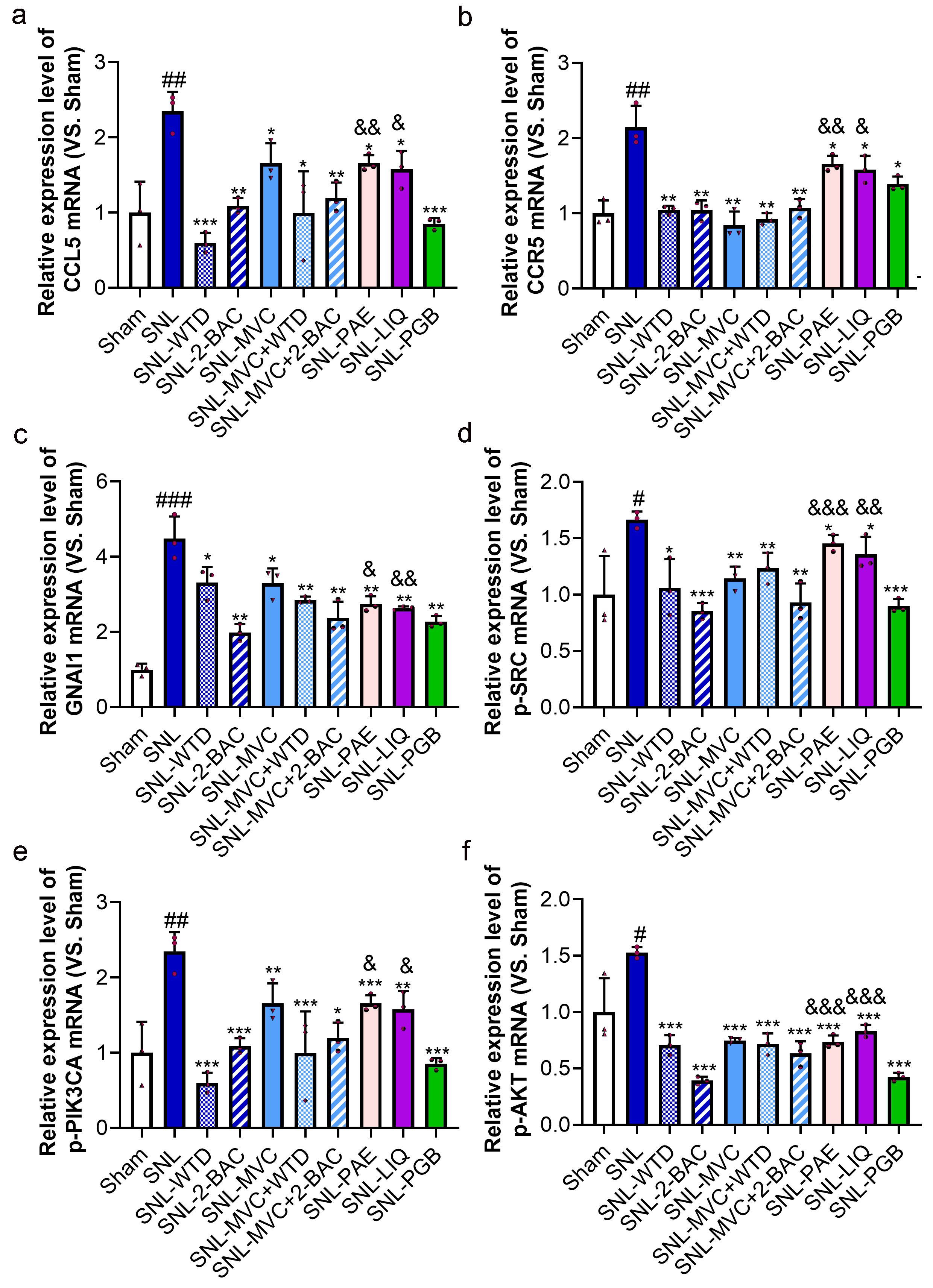

Supplement: Supplementary file 8 — Fig.S7 [file 41392_2020_160_MOESM8_ESM.tif]

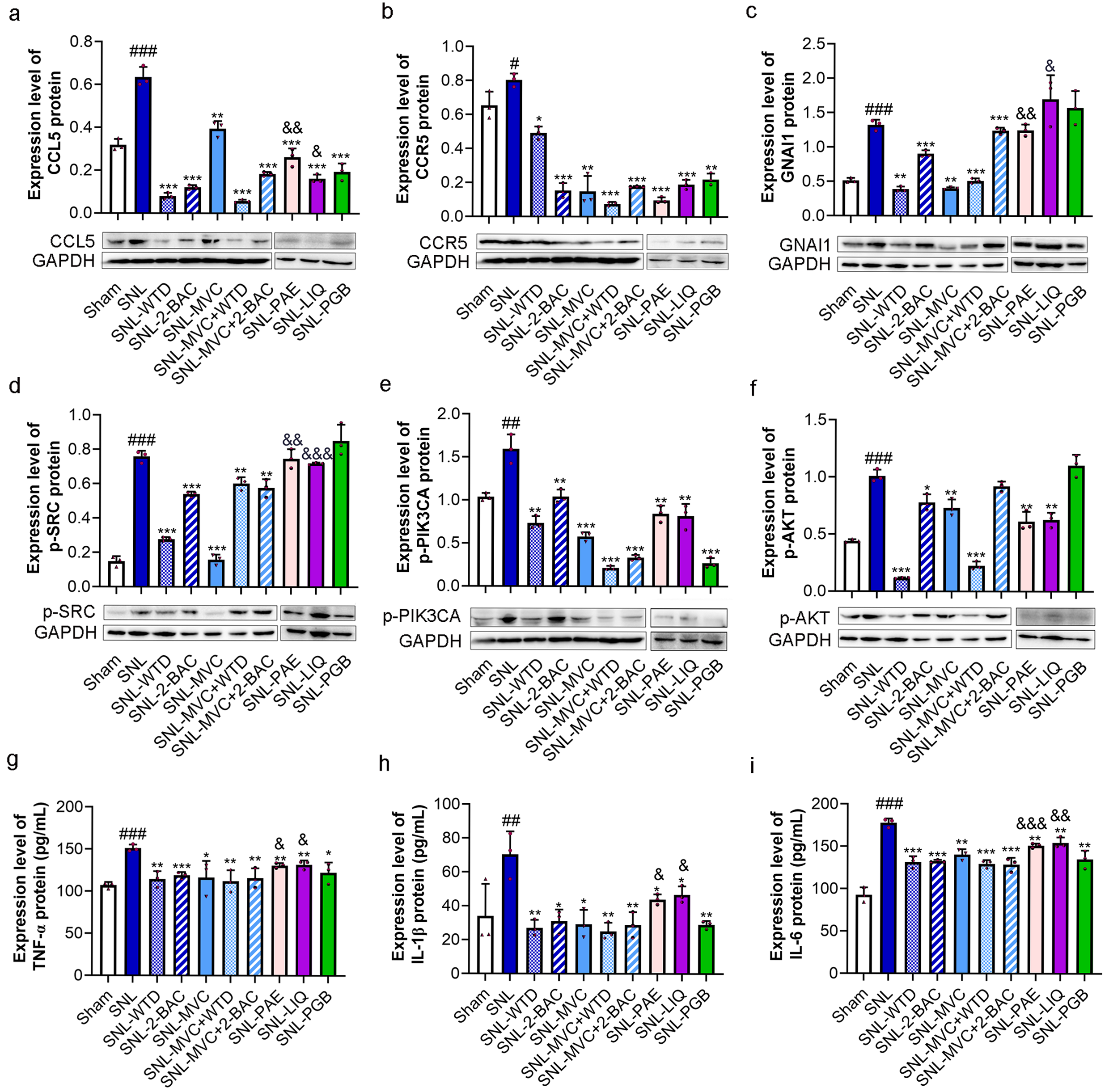

Supplement: Supplementary file 9 — Fig.S8 [file 41392_2020_160_MOESM9_ESM.tif]
