# Supplementary material for: Biomolecular network-based synergistic drug combination discovery: a combination of paeoniflorin and liquiritin alleviates neuropathic pain by inhibiting neuroinflammation via suppressing the chemokine signaling pathway
Source: Signal Transduct Target Ther. 2020 May 22;5:73. doi: 10.1038/s41392-020-0160-8 (PMC7242454; doi:10.1038/s41392-020-0160-8)
Supplement: Supplementary file 10 — raw data of Fig.S3c and Fig.S3d [file 41392_2020_160_MOESM10_ESM.docx]

Blood concentration of PAE post the administration of WTD or BAC (Fig S3c)

| Time (min) | WTD (ng/g) | | | BAC (ng/g) | | |
| --- | --- | --- | --- | --- | --- | --- |
| 5 | 44.7 | 39.9 | 75.7 |  | 489 | 847 |
| 30 | 110 | 139 | 107 | 550 | 161 | 374 |
| 120 | 133 | 146 | 152 | 294 | 131 | 137 |
| 360 | 77.7 | 146 | 70.3 | 5.21 | 4.07 | 19.4 |

Blood concentration of LIQ post the administration of WTD or BAC (Fig S3d)

| Time (min) | WTD (ng/g) | | | BAC (ng/g) | | |
| --- | --- | --- | --- | --- | --- | --- |
| 5 | 9.1 | 9.98 | 13.6 |  | 14.8 | 19.3 |
| 30 | 8.38 | 20.8 | 10.7 | 24.5 | 4 | 4.31 |
| 120 | 12.3 | 9.47 | 11.5 | 13.6 | 11.8 | 4.85 |
| 360 | 2.35 | 2.01 | 1.53 | 0.66 | 1.14 | 0.65 |
